# Nonlinear health benefits of public green space: evidence from a nationwide machine learning study in China

**DOI:** 10.3389/fpubh.2025.1680591

**Published:** 2025-10-28

**Authors:** Wei Cao, Liyan Wang, Jia Wang, Mohamed Elsadek, Deshun Zhang

**Affiliations:** ^1^Department of Landscape Architecture, College of Architecture and Urban Planning, Tongji University, Shanghai, China; ^2^College of Horticulture and Landscape Architecture, Yangzhou University, Yangzhou, China; ^3^Suzhong Development Research Institute, Yangzhou University, Yangzhou, China

**Keywords:** urban greening, public green space, self-rated health, explainable machine learning, China

## Abstract

**Introduction:**

Urban greening is widely recognized as an important factor in human health. However, existing studies have yielded inconsistent conclusions regarding its health benefits, partly due to divergent greening metrics and the prevalent assumption of linear relationships.

**Methods:**

This study investigated the associations between three types of urban greening indicators -green cover (GC), general green space (GS), and active public green space (PGS) --and the self-rated physical and mental health of urban residents across China. We matched individual-level health data from the 2020 China Family Panel Studies (CFPS) with county-level greening indicators derived from national statistical yearbooks. To account for potential nonlinearities and to evaluate feature importance, we employed explainable machine learning models (XGBoost) combined with SHapley Additive exPlanations (SHAP).

**Results:**

The results indicated that GC and GS had no significant associations with physical health, and their associations with mental health were inconsistent. In contrast, PGS and the ratio of PGS to GS (PGSRatio) demonstrated robust, significantly positive associations with both physical and mental health, with slightly stronger effects observed for physical health. SHAP-based analyses further revealed nonlinear threshold effects: PGS and PGSRatio offered limited health benefits at lower levels, but their impacts increased sharply once baseline thresholds of 12.4 and 36.3% were exceeded. Ideal health-promoting thresholds were identified at 18% for PGS and 45% for PGSRatio.

**Discussion:**

These findings emphasize that not all green space yields equivalent health benefits; rather, the provision of sufficient, accessible, and active public green space is critical for maximizing the dual health benefits of urban greening.

## 1 Introduction

According to the World Bank, the global urbanization rate reached 57% in 2023. In China, this figure has risen to 66% (National Bureau of Statistics of China, 2024), with increasing numbers of people relocating to cities in pursuit of better employment, education, amenities, and social opportunities. However, urbanization also intensifies challenges such as environmental pollution, heat stress, traffic congestion, and psychological distress, all of which adversely affect physical and mental health. Urban greening—an approach dating back to the vision of Frederick Law Olmsted (1) is widely acknowledged as a strategic intervention to mitigate these issues and promote well-being. Yet in modern urban settings, where land availability is constrained and nature access is often restricted, maximizing the health benefits of limited green resources demands evidence-based and strategic planning.

Existing studies explored the connections between urban greening and human health from three main perspectives. The most widely examined is greening quantity, where studies have investigated correlations between the amount of green infrastructure and self-reported health (2–6). A second stream of research examines the structure attributes of greening —such as vegetation type, spatial distribution, and vertical complexity—and their differential effects on health (7–9). A third focus involves the quality and usability of green spaces, including accessibility, safety, and user experience (5, 10, 11). Despite the diversity in research foci, findings remain inconsistent. While some studies report positive associations between urban greening and physical and mental health (2, 4–6), others reveal non-significant or even contextually negative effects (7, 12, 13), often shaped by factors such as air pollution, urban density, or socioeconomic status (6, 14, 15).

**Figure 1:**
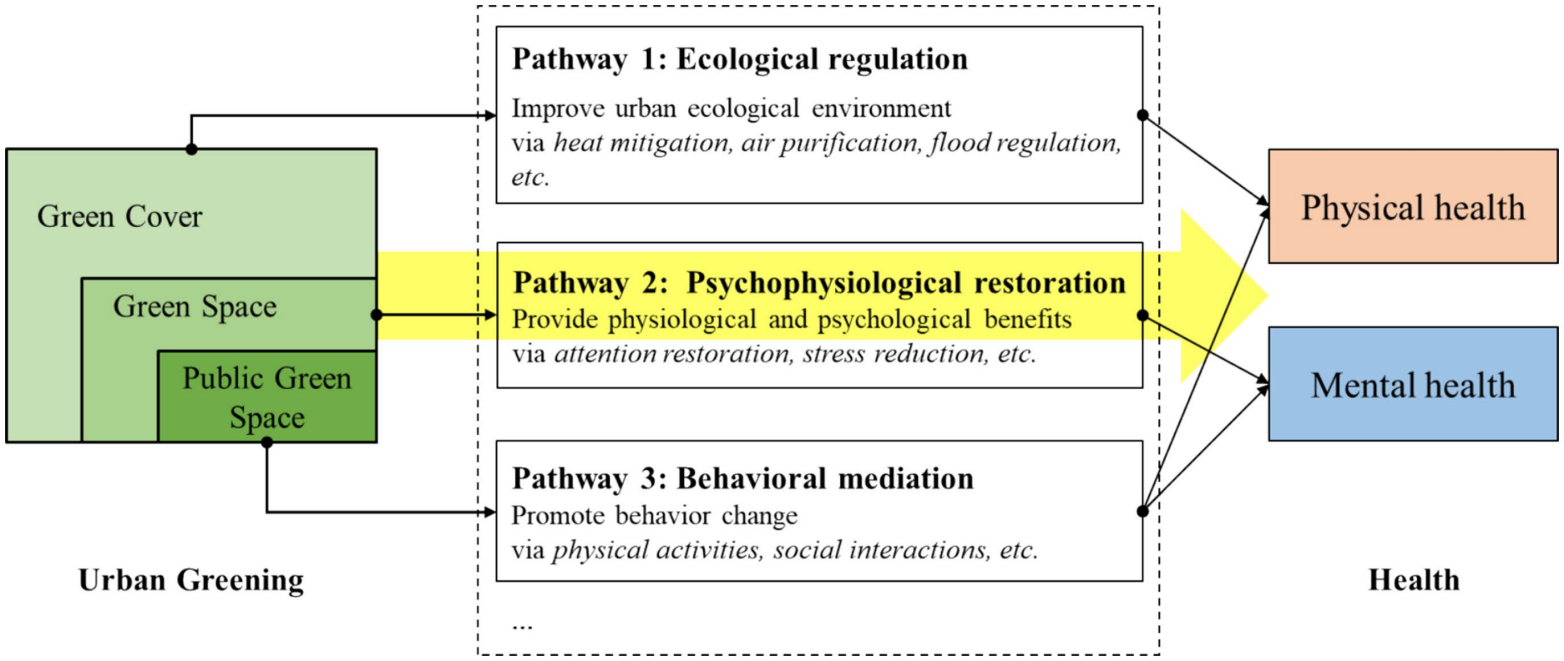
Main pathways linking urban greening to human health. Diagram adapted from Zhang et al. (16), Zhang et al. (63), and Zhang et al. (5). The yellow arrow indicates the focus of this study on quantitative urban greening indicators (GC, GS, PGS) and their dual impact on health.

Theoretically, three major pathways have been proposed to explain how greening affects human health (5, 16, 63) (see Figure 1). The first pathway, ecological regulation: vegetation contributes to cleaner air, moderated urban microclimates, and reduced environmental health risks (14, 18). The second pathway is psychophysiological restoration: natural elements provide sensory stimuli that support stress recovery, improve mood, and enhance cognitive performance (19, 20). The instantaneous, affective, and physiological responses evoked by visual stimuli can reduce stress and evoke positive emotions (21). Activities in green spaces can also facilitate sleep quality (22). The third pathway, behavioral mediation: green spaces encourage physical activity and social interaction, which confer both physical and psychological benefits (23). Being physically more active can improve cardiovascular health and reduce obesity and diabetes risks (24–26). Exercises in green spaces bring more health benefits than activities conducted in other environments, such as indoors (27, 28). Public green spaces provide venues for social interactions, encouraging frequent connections among friends and neighbors and participation in group activities (29–31). Increased social interactions are proven to benefit brain health, psychological well-being, and reduce loneliness (32).

Despite this theoretical clarity, urban greening is operationalized inconsistently across studies, contributing to contradictory findings (33). Some studies quantify green cover (GC) through remote sensing images, emphasizing horizontal vegetation extent (7), or capture vegetation health and density using NDVI (3, 6, 13). Others focus on green space (GS) derived from land-use maps, encompassing both publicly accessible and inaccessible green space (9). Still others concentrate on public green space (PGS)—green areas that are publicly accessible and recreationally functional (34). While these indicators have inclusive relationships, they each emphasize different health pathways (Figure 1): the impact of GC on health is primarily related to ecological regulating services provided by the attributes of vegetation; GS, such as parks, street trees, and green buffers, can affect health through visual and environmental exposure; while PGS is most likely to drive behavioral change due to its public accessibility and recreational functions. However, few studies have explicitly differentiated these indicators and assessed their relative importance within the same research context.

Another methodological limitation is the common assumption of linear relationships between greening and health. In reality, these associations may be nonlinear. Residents in poorly vegetated environments often suffer from compounded environmental stressors (e.g., air pollution, noise, and thermal extremes) (35), while residents in highly vegetated areas may encounter new risks, such as allergen exposure or pesticide use (36, 37). Prior research has identified nonlinear effects of residential greenery on mortality (38), respiratory illness (37), and self-rated health (3), yet few have examined such patterns for PGS specifically. A recent study in Sheffield, UK further highlighted the need for multi-indicator approaches in detecting nonlinearities related to greening and health (4).

Moreover, the interdependency between physical and mental health is increasingly recognized, involving shared physiological mechanisms across the nervous, endocrine, and immune systems (39, 40). Urban green spaces that support both physical activity and psychological restoration may thus produce compounded health benefits. Identifying thresholds or ranges at which different greening indicators yield optimal effects on both domains is an urgent empirical need but remains underexplored.

This study seeks to address these research gaps by examining the relationships between multiple urban greening metrics and self-rated physical and mental health among Chinese urban residents. Specifically, we matched self-rated health data at the individual level with urban green indicators at the county level to (1) Investigate the associations between green cover (GC), green space (GS), and public recreational green space (PGS) and residents’ physical and mental health outcomes; (2) Compare the relative importance of these metrics using explainable machine learning (XGBoost + SHAP); and (3) Identify nonlinear patterns and effective thresholds at which greening begins to exert significant dual health benefits. The findings are intended to inform green infrastructure planning, with particular attention to optimizing urban health outcomes through the strategic provision of active, accessible public green space.

## 2 Materials and methods

This study investigated the associations between urban greening and self-rated physical and mental health among Chinese urban residents. Individual-level health data were sourced from the 2020 wave of the China Family Panel Studies (CFPS) and were linked to county- and district-level greening data derived from the *China Urban Construction Statistical Yearbook 2020*. Control variables were selected at both individual and county levels, capturing personal, socioeconomic, climatic, and environmental characteristics. Data sources include CFPS, national statistical yearbooks, and remotely sensed environmental datasets. Subsections 2.1–2.3 detail the variables and data sources (see Supplementary Table S1), while subsection 2.4 describes the statistical and machine learning methods employed.

### 2.1 Self-rated health

Two dimensions of health were assessed: physical health and mental health, both based on self-rated data. Self-rated health offers several advantages over objective health measures. First, it reflects individuals’ holistic understanding of their health, incorporating illness severity, family health history, and perceived stability of health status (41, 42). Second, it demonstrates strong predictive validity for objective outcomes such as functional ability, mobility, and absenteeism, and this predictive power is consistent across socioeconomic strata (34, 43). Accordingly, self-reported health metrics are widely used in both international and Chinese population health studies (5, 34).

CFPS is a nationally representative, annual longitudinal survey of Chinese communities, families, and individuals launched in 2010 by the Institute of Social Science Survey at Peking University, China. This survey collects a wealth of information covering topics such as economic activities, education outcomes, family dynamics and relationships, migration, and health in contemporary China. The first wave of the survey encompassed 25 provinces/municipalities and 162 counties/districts in China, with more added in subsequent waves. More information about this survey can be found at https://www.isss.pku.edu.cn/cfps. This study used the individual-level questionnaire data from CFPS’s 6th wave in 2020. The County-Level Restricted Data was utilized to match individual-level data with the corresponding county data in which the respondents reside. Respondents from rural areas were excluded from the study as its primary focus was on the impact of urban greening on the health of urban dwellers.

Physical health was assessed via the question: *“How would you rate your health status?”* Responses were categorized into a binary outcome: “Excellent,” “Very good,” and “Good” were coded as 1 (good health), while “Fair” and “Poor” were coded as 0 (poor health). The dichotomization (healthy vs. unhealthy) was adopted since a statistical test indicated that the standard ordinal model was inappropriate (proportional odds assumption violated, *p* < 0.001). This binary approach followed by logistic regression reduces model complexity, avoids the need for multiple comparisons, and provides clearer results when the main research interest is in distinguishing between two groups rather than exploring all five categories. It aligns with our primary objective of identifying factors associated with the likelihood of being healthy and is widely used in similar studies (3, 5). Since this simplification could lead to information loss, we also performed OLS regression using the original 5-point variable in the third robustness test (see section 3.4 and Supplementary Table S8). Further justification for this indicator is provided in Supplementary Appendix II.

Mental health was measured using an 8-item version of the Center for Epidemiologic Studies Depression Scale (CES-D) (44), validated in the Chinese context (45). The original version includes 20 questions, and the CFPS survey used a truncated 8-item version. Among the 8 items, six items assessed negative affect and two assessed positive affect. For each item, responses indicating a more positive mental health state were given a higher score. Scores across the 8 items were summed together and a total score ranging from 8 to 32 was used to determine respondents’ health status.

### 2.2 Urban greening indicators

**Figure 2:**
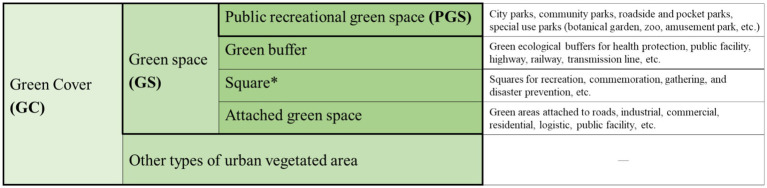
Classification of urban greening types. *Among the four types of green spaces (GS), urban squares, aside from public recreational green spaces, are also open to the public. However, they make up a very small portion of the GS area. Therefore, this study only utilized public recreational green space to represent PGS. This classification was based on the Chinese Standard for Classification of Urban Green Space CJJ/T85-2017.

We assessed four quantitative indicators of urban greening (greening within the built-up area) (Figure 2): (1) Green cover (GC): The proportion of the vertical projection area of all vegetation. (2) Green space in general (GS): The proportion of total green space, including public recreational green space, green buffers, attached green space, and squares. (3) Public green space (PGS): The proportion of green space designated for public recreational use. (4) The ratio of PGS to GS (PGSRatio): The percentage of GS that is publicly accessible (i.e., PGS as a share of GS). GS includes green spaces that are private, semi-private (such as those affiliated with gated residential communities, educational institutions, government facilities, or commercial enterprises that are only accessible to specific user groups), and public (primarily parks). In contrast, PGS is limited to fully public green spaces accessible to all. Therefore, we introduced PGSRatio to highlight the relative share of fully public green spaces. This metric enables a more comprehensive evaluation of how effectively urban greenery serves the broader population and offers insight into the equity and public utility of urban greening.

Area statistics were extracted from the *China Urban/Urban–Rural Construction Statistical Yearbook 2020*. In cases where district-level data were unavailable, values from the associated prefecture-level city were used as approximations. For interpretability, all greening indicators were multiplied by 100.

### 2.3 Control variables

This study employed control variables at both the individual and the county levels. The individual level variables comprised demographic characteristics of respondents, including age, gender, marital status, education level, individual income, and body mass index (BMI) level. Lifestyle factors were also incorporated, including cigarette smoking and alcohol consumption levels, as well as the frequency of physical activity.

The county-level variables contained socioeconomic variables, including GDP, share of secondary sector in GDP, disposable income per capita, and population density. The data on GDP, share of secondary sector in GDP, and disposable income per capita came from the China County Statistical Yearbook, and population density data was obtained from the LandScan Global Program (46). A set of climatic and environmental variables were also controlled. NDVI is a satellite-derived indicator that captures green intensity and overall plant health, reflecting the biophysical condition of vegetation. It is a valuable complement to the core urban greening variables, as the latter are yearbook-based metrics focusing on the geometric area of green spaces across various social attributes. Annual average NDVI of each county was calculated based on MOD13A3.^1^ DEM data were derived from NASA Shuttle Radar Topography Mission (SRTM)^2^ (47). Relative humidity and mean temperature data were acquired from the National Tibetan Plateau/Third Pole Environment Data Center (48, 49). PM_2.5_ concentration data were obtained from ChinaHighAirPollutants (CHAP) datasets^3^ (50). All remote sensing data were at a spatial resolution of 1 × 1 km. The annual mean data of each county were calculated on the ArcGIS platform.

### 2.4 Data analysis

#### 2.4.1 Statistical analysis

Three stages of statistical analysis were performed: (1) Spearman’s rank correlation was used to examine relationships among variables, chosen for its suitability with non-parametric and discrete data. (2) Binary logistic regression was applied to assess the relationship between physical health and the four greening indicators, adjusting for all control variables. Outputs include odds ratios (OR), *p*-values, standard errors, and 95% confidence intervals (CI). (3) Ordinary Least Squares (OLS) regression was used for the 25-level mental health outcome, with unstandardized coefficients and corresponding inferential statistics reported. OLS model was chosen over ordinal logistic regression model since the proportional odds assumption was violated, making ordinal regression model inappropriate. For both regression approaches, individual cross-sectional weights were applied to ensure national representativeness, and standard errors were clustered at the county/district level to account for intra-group correlation. All statistical analyses were conducted using Stata 18.0.

#### 2.4.2 Machine learning: XGBoost and SHAP

This study employed the eXtreme Gradient Boosting (XGBoost) model in conjunction with SHapley Additive exPlanations (SHAP) to assess the relative importance of urban greening indicators and to visualize specific associations with self-rated physical and mental health outcomes.

XGBoost is a scalable and high-performance machine learning algorithm that implements a gradient boosting by constructing an ensemble of sequential, shallow decision trees (51). This ensemble approach improves predictive accuracy by allowing each tree to iteratively correct the errors of its predecessors. XGBoost is particularly advantageous due to its capacity for parallel computation, robust handling of missing data, sensitivity to outliers, and effective performance on small and complex datasets. When appropriately tuned, it frequently outperforms comparable algorithms, such as random forests (52). The application of XGBoost has been increasingly adopted in environmental health research, including studies on the effects of the urban environment on health outcomes (53, 54).

In this study, classification models were used to analyze binary physical health outcomes, while regression models were employed for mental health scores. We conducted hyperparameter tuning for the XGBoost models using a grid search with 5-fold GroupKFold cross-validation to account for the clustered data structure at the county level. This ensured that all observations from the same county were assigned to a single fold, either entirely in training or entirely in testing, thereby preventing data leakage and providing robust performance estimates. Hyperparameter tuning was performed exclusively on the training set. The grid search evaluated a predefined set of candidate parameters designed to balance model complexity and prevent overfitting. The optimal hyperparameters identified through this procedure were: colsample_bytree = 1.0, gamma = 0, learning_rate = 0.01, max_depth = 3, n_estimators = 200, subsample = 0.8, and scale_pos_weight = 1. Model performance was evaluated using the following metrics: (1) For classification (physical health): accuracy, precision, recall, and F1-score, ROC-AUC, and PR-AUC; (2) For regression (mental health): mean squared error (MSE), root mean squared error (RMSE), mean absolute error (MAE), mean explained variance (MEV), and coefficient of determination (R^2^).

While XGBoost provides high predictive performance, its internal decision-making process is often described as a “black box” due to its complexity. To enhance interpretability, the SHAP framework was employed. SHAP is grounded in cooperative game theory and uses Shapley values to estimate the marginal contribution of each feature to the model’s output (55). It offers advantages over other interpretation techniques by providing locally accurate, consistent, and model-agnostic explanations tailored to individual predictions. The Shapley value of feature *i* is calculated using Equation 1:


(1) $$\emptyset_{i}=\sum_{S\subseteq N\{i\}}\frac{|S|!(n-|S|-1)!}{n!}[f(S\cup \{i\})-f(S)]$$

where $\emptyset_{i}$ represent the contribution of the *i*th feature, *N* stands for the dataset with *n* features, *f*(*S* ∪{i} ) and *f*(*S*) denote the model results with or without feature *i*, respectively. Then an additive feature imputation approach was employed to calculate the SHAP value using Equation 2:


(2) $$g(z\prime )=\phi_{0}+\sum_{i=1}^{M}\phi_{i}z\prime$$

where *g* represents the interpretation model; *z*’∈{0,1}*^M^* denotes whether a feature is present (*z*’ = 1) or not (*z*’ = 0) in the calculation, and *M* is the number of input features; $\phi_{0}$ is the base value and $\phi_{i}$ is the Shapley value of feature *i*.

All the analyses were conducted in the secured server laboratory of the Institute of Social Science Survey, Peking University, Beijing, China.

## 3 Results

### 3.1 Descriptive statistics

We matched individual-level self-rated health data from the CFPS2020 with county- and district-level urban greening and control variables. The final analytical sample comprised 12,854 urban respondents from 625 counties and districts, covering 197 prefecture-level cities across 22 provinces and 4 centrally governed municipalities in China. The geographic matching success rate was 98.6%, excluding all rural samples.

#### 3.1.1 Self-reported health

**Figure 3:**
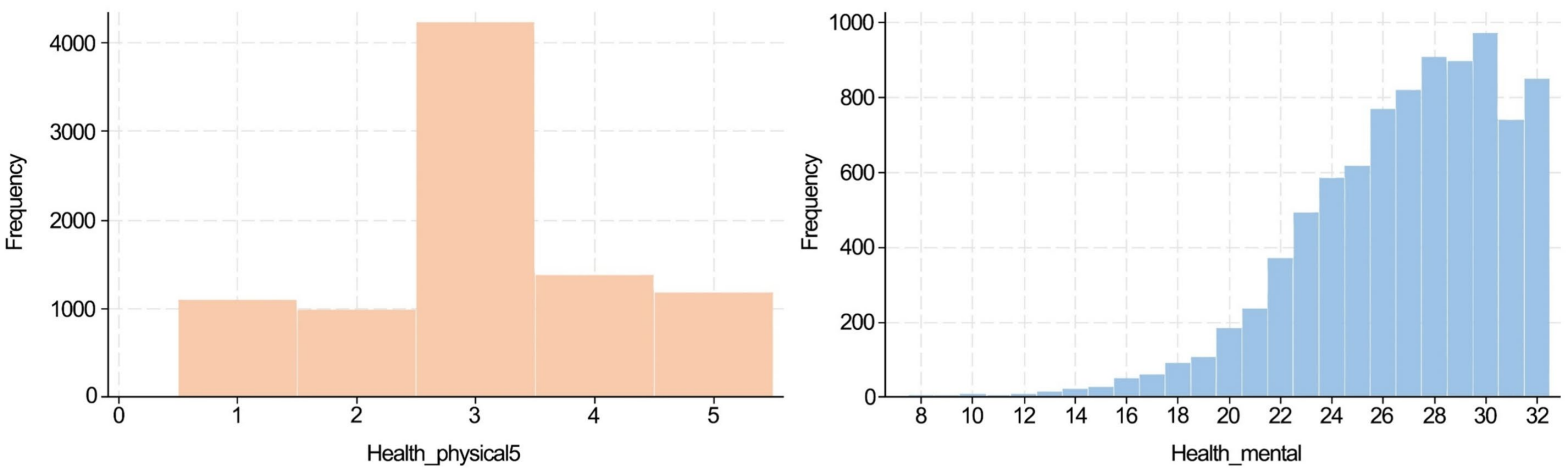
Data distribution of physical and mental health.

Due to missing responses on key health variables, the final sample sizes used in regression models were smaller than the total matched dataset. Summary statistics are presented in Table 1 and Figure 3. The physical health was assessed on a five-point ordinal scale. The distribution was approximately symmetrical, with the largest proportion of respondents 48%, assigning themselves a score of 3 (midpoint). The proportions of respondents rating their health as 1, 2, 4, and 5 were similar, each around 11–15%. Overall, according to our binary classification, approximately 75% of respondents considered themselves as being in good health conditions, while the remaining 25% reported poor health conditions.

**Table 1:** Descriptive statistics.

Variables	Obs	Mean	SD
Dependent variable			
Health_physical	8,898	0.765	0.424
Health_mental	8,861	26.809	3.914
Independent variable			
GC	8,538	38.966	6.683
GS	8,272	34.916	6.966
PGS	8,897	11.036	4.458
PGSRatio	8,272	33.121	13.530
Control variable_individual level			
Age	8,898	46.966	15.695
Gender	8,898	0.491	0.500
Education	8,898	9.899	4.611
Marriage	8,898	0.816	0.387
Income	8,898	2.879	1.008
Smoke	8,898	0.263	0.441
Drink	8,898	0.133	0.340
Exercise	8,898	2.047	2.497
Overweight	8,898	0.097	0.296
Underweight	8,898	0.059	0.236
Control variable_county-level			
lnGDP	8,898	15.132	1.361
PropofIndustry	8,898	0.341	0.154
lnDisposableIncome	8,898	10.581	0.316
lnPopDens	8,898	6.607	1.595
DEM	8,898	459.353	634.561
RH	8,898	0.712	0.083
TempAve	8,898	14.466	5.117
PM25	8,898	34.071	10.914
NDVI	8,898	0.466	0.124

The minimum, maximum, and median values of the variables are not provided due to confidentiality requirements set by CFPS for the County-Level Restricted Data. Disclosure of this information may enable readers to identify specific counties or districts, which are subject to strict restrictions imposed by CFPS.

The mental health was measured using the 8-item CES-D scale, with total scores ranging from 8 to 32. The mean score was 26.8 (SD = 3.9), and the distribution was left-skewed, indicating that most respondents perceived themselves to be in good mental health conditions. The scale demonstrated acceptable internal consistency (Cronbach’s alpha value = 0.775), confirming its reliability in the present sample. The correlation between self-rated physical and mental health was statistically significant but modest (Spearman’s rho = 0.209, *p* < 0.01), indicating that the two measures reflect related but distinct health dimensions.

We further conducted Spearman’s rank correlation analyses to examine the correlation between personal characteristic variables (Supplementary Table S2), county-level variables, and self-rated health (Supplementary Table S3). The results indicated that most variables were significantly but weakly correlated with health, with age and education showing slightly stronger correlations with physical health. Most county-level variables were also weakly but significantly correlated both health dimensions.

Due to CFPS confidentiality policies, address-level information below the provincial level (i.e., prefecture, county, and district) is anonymized. Consequently, spatial distribution maps of respondent locations and associated greening or health data are not provided in this study.

#### 3.1.2 Urban greening indicators

As shown in Table 1, the mean values for the four urban greening indicators were as follows: green cover (GC) = 39.0%, green space (GS) = 34.9%, public green space (PGS) = 11.0%, and public green space ratio (PGSRatio) = 33.1%. These values suggest that, on average, GC and GS occupied a similar proportion of the built-up urban area, while only approximately one-third of the total green space was publicly accessible and recreationally functional.

The Spearman correlation analysis (see Supplementary Table S4) revealed a strong positive correlation between GC and GS, indicating that urban areas with greater vegetative cover also tend to report more total green space. PGS was moderately correlated with both GC and GS, implying partial overlap but not equivalence. In contrast, PGSRatio showed minimal correlations with GC and GS, suggesting that the overall quantity of vegetation or green space is not predictive of the proportion of space that is accessible to the public. Due to the confidentiality protocols of the CFPS dataset, detailed distributions (e.g., minimum, maximum, and spatial variations) of these greening indicators at the county or district level are not disclosed in this study.

### 3.2 Relationships between urban greening indicators and self-rated physical health

We examined the associations between each of the four urban greening indicators and self-rated physical health by constructing four separate binary logistic regression models [Model (a)–(d)]. Each model included one greening indicator as the core independent variable, along with a set of covariates. The results are presented in Table 2 and detailed in Supplementary Table S5. Variance Inflation Factors (VIFs) for these four models are between 2.10–2.30. The analysis revealed that GC and GS were not significantly associated with physical health status. By contrast, PGS and PGSRatio demonstrated statistically significant positive effects.

**Table 2:** Associations of urban greening indicators with physical and mental health.

Adjusted models*	Core variables	Coef.	Odds ratio	*p*-value	95% CI	
					Upper	Lower
Physical health						
Model (a)	GC	0.004	1.004	0.344	0.995	1.014
Model (b)	GS	0.006	1.006	0.177	0.997	1.015
Model (c)	PGS	0.025	1.025	0.000	1.013	1.039
Model (d)	PGSRatio	0.006	1.006	0.015	1.001	1.010
Mental health						
Model (e)	GC	0.008		0.265	−0.006	0.023
Model (f)	GS	0.008		0.297	−0.007	0.022
Model (g)	PGS	0.021		0.022	0.003	0.039
Model (h)	PGSRatio	0.008		0.012	0.002	0.014

*Adjusted for Age, Gender, Education, Marriage, Income, Smoke, Drink, Exercise, Overweight and Underweight at the individual level, and lnGDP, PropofIndustry, lnDisposableIncome, lnPopDens, DEM, RH, TempAve, PM2.5, and NDVI at the county level.

Specifically: A 1% increase in PGS within the built-up urban area was associated with an approximately 2.5% increase in the odds of respondents reporting good physical health, after adjusting for all control variables. A 1% increase in PGSRatio was associated with a 0.6% increase in the odds of self-reported good physical health. These results highlight the importance of public accessibility and recreational functionality in green space provision, beyond the mere presence of vegetative or green-covered land.

To further examine and validate the relative importance of urban greening indicators in predicting physical health, XGBoost classification models were employed, coupled with SHAP values for model interpretation. Given the high correlations between GC and GS and between PGSRatio and PGS (Supplementary Table S4), we constructed two models. Model I included GS and PGS to assess their relative importance, which helped verify the robustness of the results (that PGS is more critical than GS) and to capture any nonlinear relationships with health. Model II incorporated PGSRatio alone to further examine the nonlinear association between the share of fully public green space within general green space and health outcomes. The models’ performance was evaluated using standard classification metrics. The models achieved an accuracy of 0.76, precision of 0.80, recall of 0.91, F1-score of 0.85, ROC-AUC of 0.72, and PR-AUC of 0.89, indicating satisfactory predictive performance. While accuracy only slightly exceeds the majority-class baseline of 75%, the higher precision, recall, and PR-AUC demonstrate that the model provides added predictive value beyond trivial classification.

**Figure 4:**
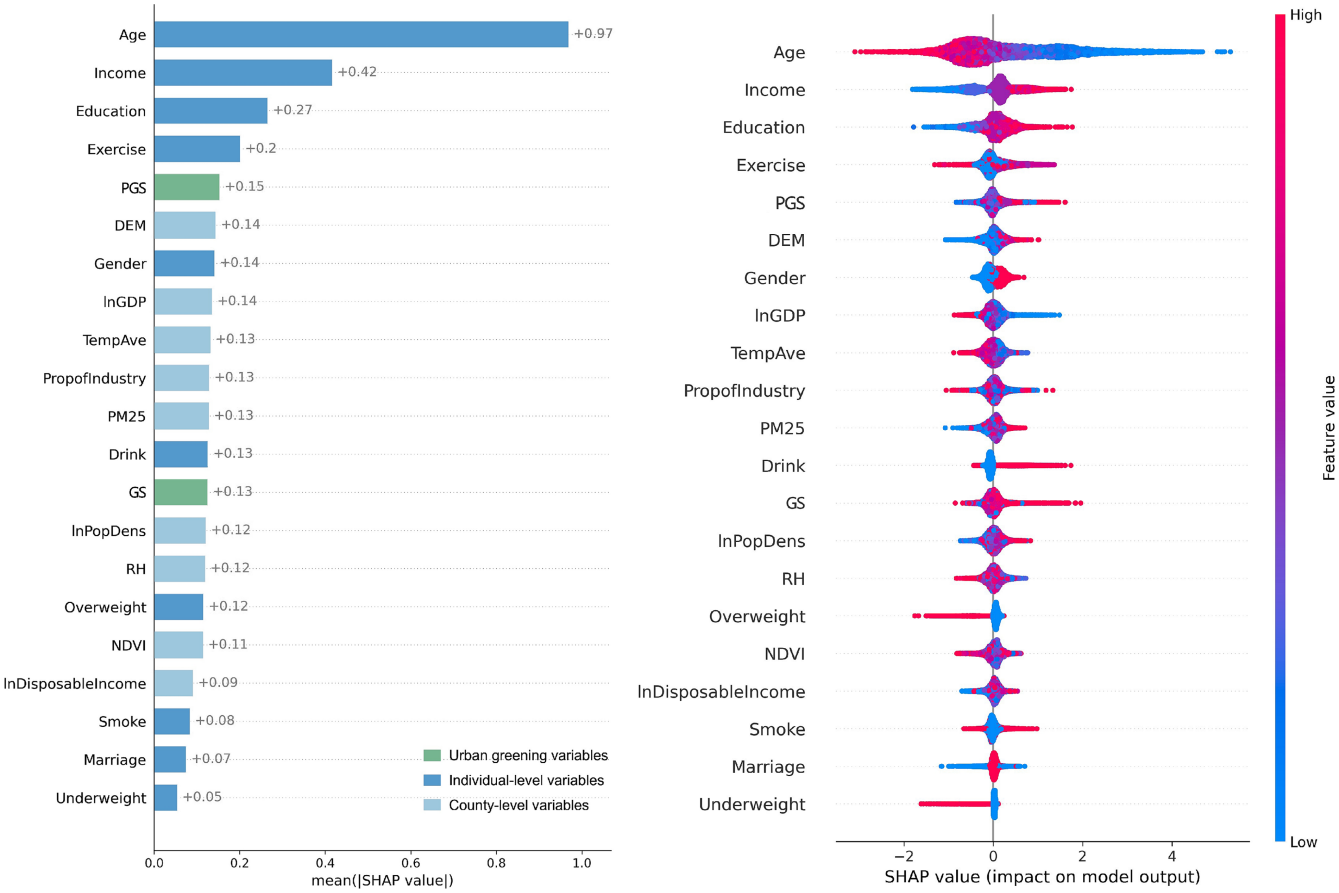
Relative importance of GS and PGS on physical health from Model I.

The relative importance of the variables is presented in Figure 4. PGS was the 5th most important variable for physical health among all the variables examined, whereas GS ranked 13th. This indicated that PGS had a stronger influence on physical health outcomes. At the individual level, age was the most influential predictor, followed by income, education, and physical activity. Among county-level variables, elevation (DEM) and average temperature were the most important climatic predictors, while GDP and industrial proportion were the most relevant socioeconomic variables. All of these were less influential than PGS.

**Figure 5:**
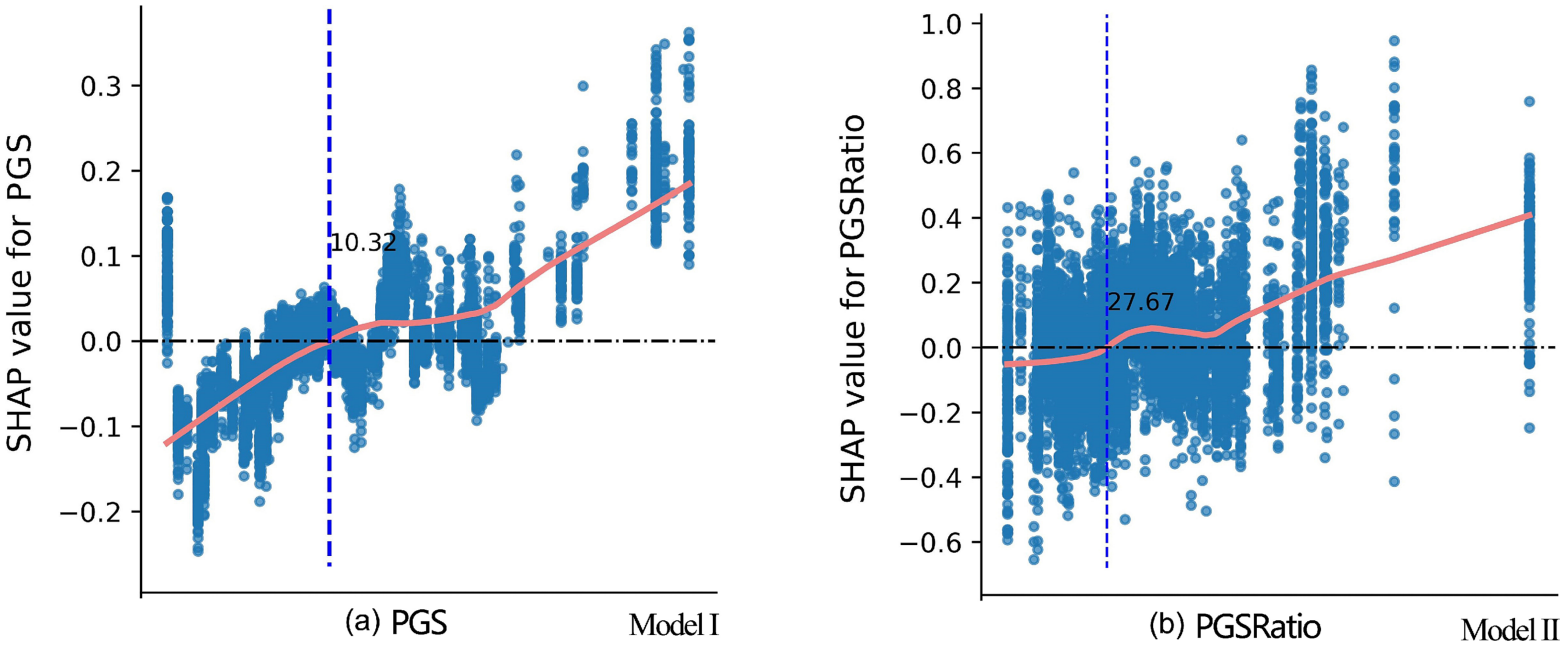
SHAP dependence plots for PGS and PGSRatio from Model I and Model II, respectively. The marks of the x-axis are not provided due to confidentiality requirements set by CFPS for the County-Level Restricted Data. Disclosure of this information may enable readers to identify specific counties or districts, which are subject to strict restrictions imposed by CFPS. The approximate turning point values for PGS (18%) and PGSRatio (45%) were provided by CFPS staff who can actually see the marks on x-axis.

The nonlinear patterns and threshold effects were examined using SHAP dependence plot (Figure 5). In these plots, the x-axis represents the actual value of PGS and PGSRatio, which are the two variables that significantly impact physical health. The y-axis represents the SHAP values that the selected features contribute to the outcomes. A higher SHAP value represents a greater positive contribution of the variable to the model prediction results. A fitted curve (Locally Weighted Scatterplot Smoothing, LOWESS) was used to smooth the scatterplot. The steeper the curve, the higher the marginal effect of the independent variable. From Figure 5A, a positive relationship is observed between PGS and physical health. When PGS was smaller than 10.3%, PGS exhibited a negative contribution to the prediction of good physical health. Specifically, low PGS had negative contributions to physical health, and this negative contribution gradually diminished as PGS increased. When PGS reached 10.3%, the contribution of PGS to physical health became neutral, indicating no additional impact. As PGS further increased beyond this threshold, it began to show positive influence, albeit with some fluctuations. Notably, when PGS exceeded approximately 18%, it demonstrated a strong positive impact on physical health. In Figure 5B, a higher level of PGSRatio is associated with better physical health. When PGSRatio was below 27.7%, the SHAP values increased slightly and fluctuated around 0, indicating that PGSRatio had a minimal impact on physical health within this range. However, once PGSRatio exceeded the threshold of approximately 45%, it began to exert a strong influence on physical health.

### 3.3 Relationships between urban greening and self-rated mental health

Ordinary least squares (OLS) regression models, Model (e)–(h), were conducted to examine the associations between each urban greening indicator and self-rated mental health. The results are summarized in Table 2. VIFs for these four models are between 2.10 and 2.30. In line with the physical health findings, GC nor GS were not significantly associated with mental health. However, both PGS and PGSRatio showed statistically significant positive associations. A 1% increase in PGS was associated with a 0.021-point increase in the mental health score, controlling for all covariates. A 1% increase in PGSRatio corresponded to a 0.008-point increase in the mental health score.

Using the same analytical approach as section 3.2, we constructed Model III, which included GS and PGS, to compare their relative importance, and Model IV, which incorporated PGSRatio, to demonstrate how changes in it affected mental health. The evaluation metrics for the models—MSE, RMSE, MAE, and R^2^—were 14.57, 3.82, 3.06, and 0.08, respectively. Although the R^2^ value was relatively low, it remained comparable to other studies examining the same relationship (5, 6, 17). Among these studies, the R^2^ values from the questionnaire studies were slightly higher due to better alignment between the dependent and independent variables.

**Figure 6:**
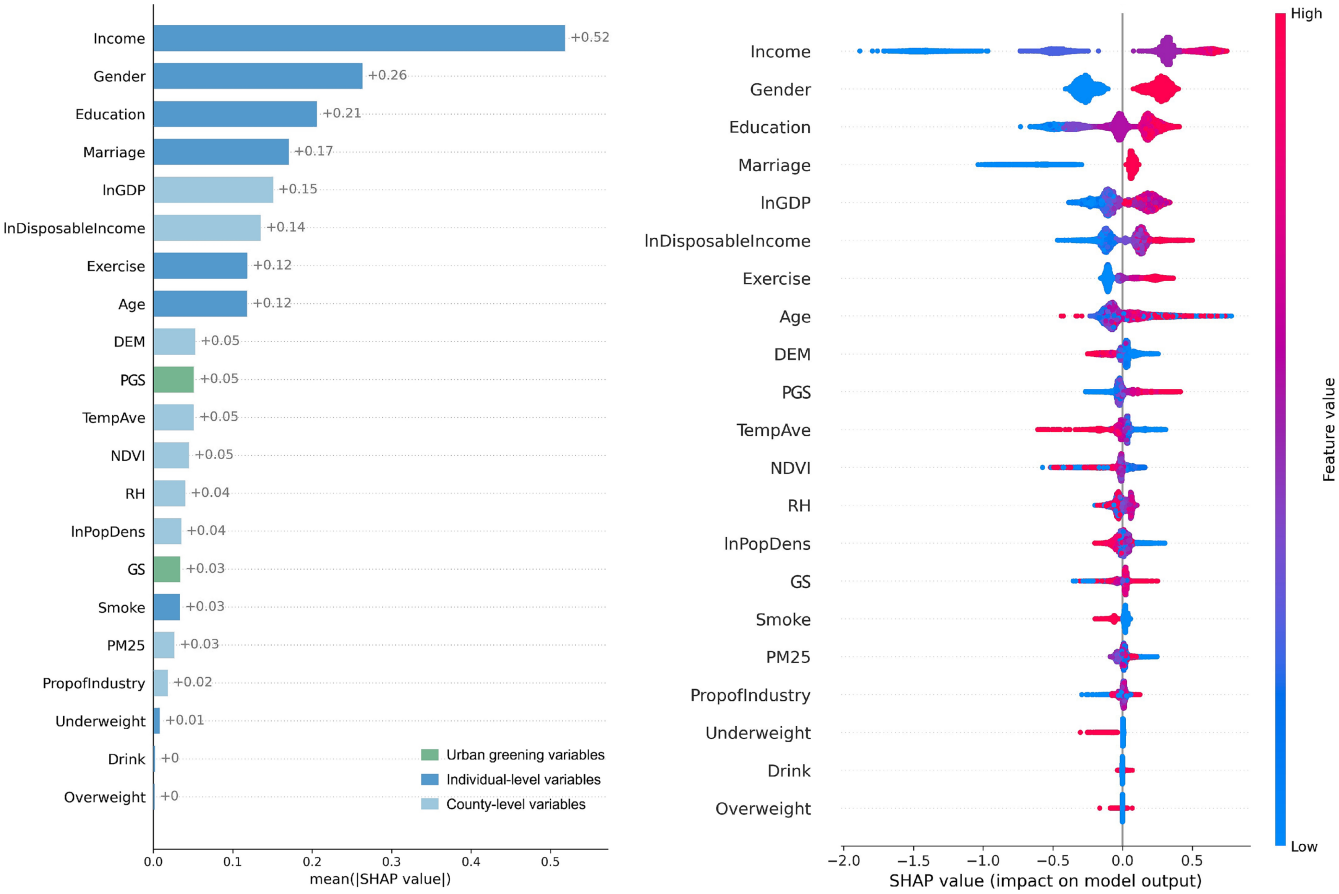
Relative importance of GS and PGS on mental health from Model III.

The results (Figure 6) revealed that PGS ranked 10th and GS ranked 15th in affecting mental health, indicating that PGS had a greater impact than GS. Personal characteristics had the most significant impact on mental health. Residents with higher incomes, who were male, had a higher level of education, and were in a relationship tended to exhibit better mental health conditions. Socioeconomic factors at the county level, represented by GDP and disposable income, were more critical to mental health than climate and environmental factors. Overall, PGS had less impact than these factors.

**Figure 7:**
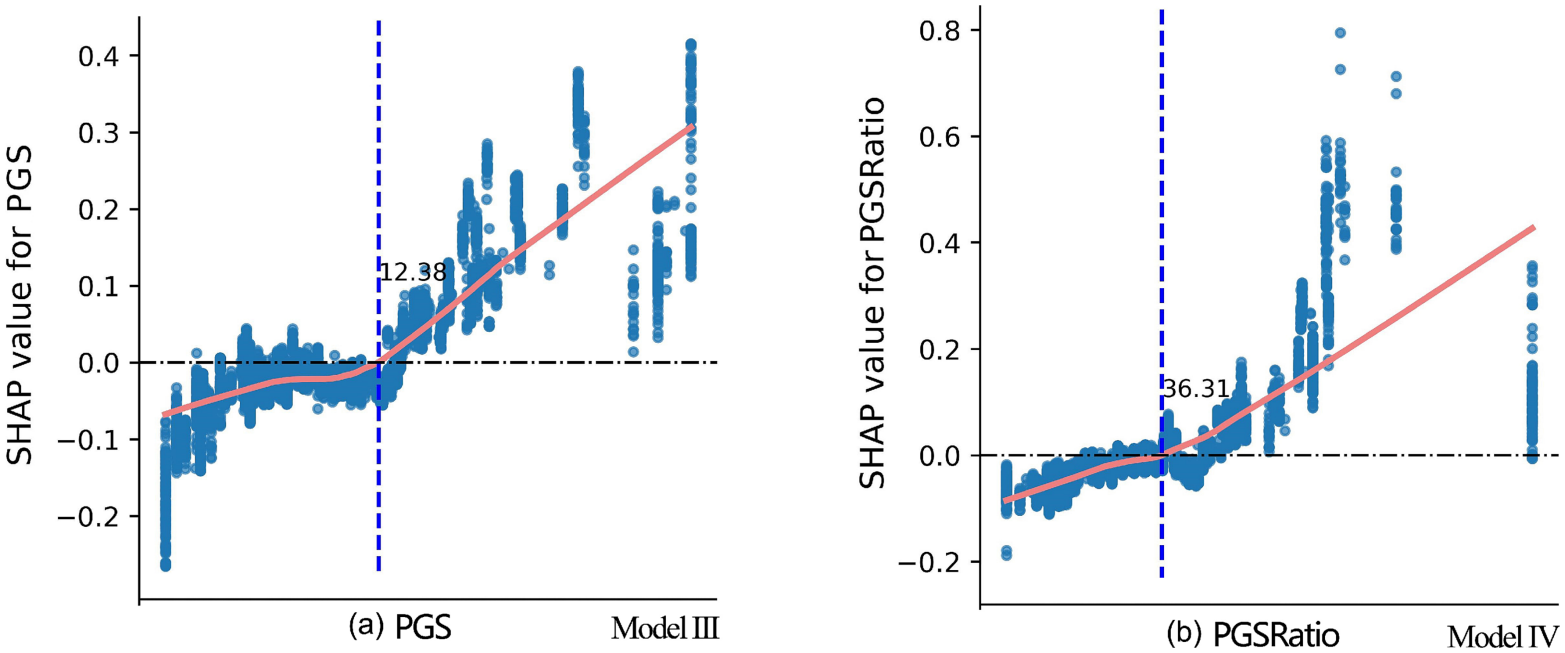
SHAP dependence plots for PGS and PGSRatio from Model III and Model IV, respectively. The marks of the x-axis are not provided due to confidentiality requirements set by CFPS for the County-Level Restricted Data. Disclosure of this information may enable readers to identify specific counties or districts, which are subject to strict restrictions imposed by CFPS.

As shown in Figure 7A, an overall upward trend in mental health scores is observed with PGS. However, when PGS was less than 12.4%, this trend manifested as gradual and fluctuating. The SHAP values were close to 0, suggesting that the contribution of PGS to mental health prediction was minimal within this range. When PGS exceeded 12.4%, a substantial positive impact on mental health was evident. The analysis of PGSRatio (see Figure 7B), revealed that when PGSRatio was less than 36.3%, its contribution to mental health was minor and negative, and this negative contribution decreased as its value increased. When PGSRatio exceeded 36.3%, it exerted a significant positive effect on mental health.

### 3.4 Robustness tests

To confirm the robustness of the main findings, five supplementary tests were conducted. Detailed results are provided in Supplementary materials. First, all continuous variables were winsorized at the 5th and 95th percentiles. The re-estimated logit and OLS models (Supplementary Table S6) produced results that were consistent with the baseline models (Supplementary Table S5), indicating that extreme values did not influence the observed associations. Second, a new control variable, SunshineHours, was introduced to account for the potential influence of solar exposure on health outcomes. The results (Supplementary Table S7) showed that the significance of PGS increased, reinforcing its contribution to both physical and mental health. Third, to examine the sensitivity of results to model choice, we applied OLS regression to the physical health outcome using the original five-point scale (Health_physical5), and logit regression to a binary mental health variable (Health_mental01, coded as 1 for scores 21–32, and 0 for scores 8–20). While these model forms are not ideal—given the ordinal structure of Health_physical5 and the left-skewed distribution of Health_mental01—the results (Supplementary Table S8) continued to show that PGS and PGSRatio remained more strongly associated with health outcomes than GC or GS. Fourth, to further test robustness, we replaced the binary self-rated health outcome with an objective measure, Health_hospitalized, indicating whether the respondent had been hospitalized in the past 12 months. Logit model results (Supplementary Table S9) showed that PGS and PGSRatio were significantly negatively associated with hospitalization, providing additional support for their relevance to physical health. No suitable alternative indicator for mental health was available in the CFPS dataset. Finally, the full analysis was repeated using the 2018 wave of CFPS. Results (Supplementary Table S10) confirmed the original findings: PGS and PGSRatio had significant positive effects on physical health, while GC and GS remained non-significant. For mental health, however, all four greening indicators were positively associated. Nonetheless, the XGBoost model comparing GS and PGS (Supplementary Figure S1) continued to show that PGS had greater predictive importance, aligning with the primary analysis.

## 4 Discussion

### 4.1 Contribution of urban greening to physical health

This study found that GC and GS were not significantly associated with self-rated physical health, whereas PGS and PGSRatio had consistently significant positive impacts. These findings indicate that the presence of vegetation alone is insufficient; instead, accessible, well-designed public green spaces are more critical to supporting physical health outcomes in urban populations.

Potential reasons for the findings are discussed based on the pathways presented in Figure 1. Regarding the first pathway, the positive effect of GC or GS on physical health through improving the ecological environment is uncertain across studies. For example, Jaafari et al. (8) and Bi et al. (2) revealed that GC and GS significantly promoted physical health by reducing air pollution, whereas Fu et al. (14) found that the health benefits of GS were negligible in regions with significant air pollution risks but pronounced in areas free from such risks across China. Song et al. (13) observed no significant modification effect of GC on heat-related mortality risk in Hong Kong. These inconsistencies underscore the complexity of the ecological regulation pathway, which may be deeply influenced by climatic, geographic, or other regional conditions.

In the second pathway, the physiological responses may contribute to physical health. However, Zhang et al. (5) found that visual greening had no significant effect on general health, which might be due to the fact that the physiological responses caused by visual stimuli were usually short-term (5, 20). Furthermore, Veitch et al. (56) emphasized that it is the act of physical exercise, rather than the environment in which it occurs, that drives acute physiological responses. This implies that the provision of active public green spaces for physical activity plays a more critical role in generating health benefits than the mere presence of vegetation.

The third pathway pertains to the promotion of healthy behavior, which is best realized through easily accessible and well-designed public green spaces. Two questionnaire-based studies confirmed that public green spaces play an important role in promoting physical activity and social interactions (29, 57), which directly promote physical health. Ordinary vegetated areas or certain types of urban green spaces, such as green buffers or attached green spaces, may not provide sufficient public access or recreational opportunities to fulfil such functions (53, 58). Although the literature on PGS and population health is still limited, this study confirmed the relationship and identified specific quantitative thresholds.

### 4.2 Contribution of urban greening to mental health

The results showed that the effects of GC and GS on mental health were non-significant when employing 2020 data, yet they exhibited significance when utilizing 2018 data, suggesting a lack of stability. In contrast, the effects of PGS and PGSRatio on mental health were significant across both years and all robustness tests, with PGS demonstrating a stronger influence compared to GS.

Previous studies have also documented inconsistency associations between GC and GS and mental health. The prevailing assumption is that abundant greening benefits mental health compared to less abundant greening conditions. This has been supported by many earlier studies (5, 19, 59). However, a systematic review conducted by Gascon et al. (12) showed limited evidence of the long-term benefits of surrounding greening on mental health. Ha et al. (7) discovered that the quantity of GC had no significant impact on psychological distress; whereas its spatial configuration did. Furthermore, Tomita et al. (15) found that higher NDVI was predictive of better mental wellbeing only among middle-income groups. Zhu et al. (6) reported that urban-scale greening was associated with improved mental health, but only in mid-sized and mid-density cities.

Urban greening affects mental health mainly through the second and third pathways outlined in Figure 1. The second pathway pertains to the visual stimuli from green environments, which generates beneficial physiological and psychological responses. However, first, the mental health benefits from urban greening might be temporary or short-term (19, 20). Second, the benefits may be weak compared with other factors that have a more direct and stronger influence on mental health, such as struggles in career or family relationships, loneliness and isolation, sleeping problems, etc. Third, simply having green cover or green space does not ensure visual or psychological appeal. For instance, commonplace street trees or routine landscaping around buildings, though frequently encountered, may lack aesthetic quality or mental restoration benefits needed to enhance well-being. Therefore, results from studies examining the impact of GC or GS on mental health are not consistent.

This study proceeded to explore the impact of the public portion of green space on mental health. The significantly positive and robust result verifies the importance of the third pathway, which requires enough, easily accessible, and well-designed public open space to stimulate social interactions and physical exercise, thereby fostering mental well-being. Although there are few directly comparable studies, questionnaire studies using respondents’ personal experiences with green space (which means in public green spaces) usually draw the same conclusion. For example, Zhang et al. (5) and Zhu et al. (11) found qualitative attributes of public green spaces, such as perceived usage, activity, environmental quality, amenity, and safety, were positive for mental well-being. Our study advances the literature by identifying a specific quantitative threshold of PGS that facilitates population-level mental health.

### 4.3 Consistencies in the effects of urban greening on physical and mental health

Two key consistencies were identified regarding the health effects of urban greening. First, PGS had more pronounced effects than GC and GS on both physical and mental health. This implies that promoting behavioral change is the core mechanism linking urban greening to health outcomes. Encouraged behaviors, such as physical activity and social interaction, yield both physical and psychological benefits. This dual effect may be attributed to the close relationship between physical and mental health, mediated through shared physiological mechanisms including endorphin release, stress reduction, and modulation of inflammatory responses (39, 40, 60).

Second, both PGS and PGSRatio, exhibited similar nonlinear relationships with physical and mental health. When PGS levels were below 10–12% and PGSRatio fell below 28–36%, their health benefits were very minimal. A possible explanation is that limited PGS within a county or district often results in spatial configurations that are either small and dispersed, or large and concentrated. In the former, design quality, accessibility, and amenities may be compromised, discouraging use. In the latter, even well-designed green spaces may be distant and thus less frequently visited. As PGS and PGSRatio increase (to 18 and 45%, respectively), these spatial constraints diminish, allowing benefits to accrue more consistently and equitably.

### 4.4 Endogeneity concerns and other limitations

This cross-sectional study may be subject to endogeneity issues (61), primarily through simultaneity and omitted variable bias. Simultaneity, or reverse causality, may occur if health-conscious individuals preferentially settle in greener districts. However, prior research indicates that employment, housing, and education are more influential in urban settlement decisions in China (62). The quantity of public green space likely plays a lesser role, suggesting limited threat from reverse causality.

In terms of omitted variable bias, the health of individuals and communities is affected by a wide range of complex factors, including the physical, economic, social, and behavioral determinants. While we controlled for multiple individual- and county-level variables (e.g., income, education, pollution, and climate), unmeasured factors such as genetic predisposition, occupational stress, or healthcare access could influence outcomes. This complexity helps explain why the models exhibit relatively low explanatory power. Nonetheless, unless these omitted variables are also correlated with greening levels, they are unlikely to severely bias our estimates.

Other than the potential endogeneity issues, this study has several other limitations. First, it matched the microdata on respondents’ self-rated health with the meso-data on the greening levels in the respondents’ counties or districts, and concluded that PGS is crucial to well-being. However, the amount of time spent in public green spaces and the way in which they interact with them are different for each respondent. For instance, older adults and children might spend more time in public green spaces compared to middle-aged working adults. Therefore, our results may be strengthened or weakened due to these confounders. Future research could incorporate questionnaires to directly capture the roles of green space in respondents’ lifestyle, including time spent in green spaces, types of activities, etc., to make the findings more accurate.

Second, this study identified only the quantitative importance and effective thresholds for public green spaces. Future studies should prioritize the quality of them by integrating spatial patterns (compositional and configurational features), user experience (accessibility, amenity, aesthetics, safety, etc.), and equity in providing PGS across different demographic and socioeconomic groups.

Third, one key limitation of this study lies in the treatment of *PM_2.5_*, *Exercise*, and *Overweight/Underweight* as control variables. These factors may act as mediators through which greenspace indirectly affects health by encouraging physical activity, reducing obesity, or improving air quality. Their inclusion may attenuate the estimated total effects of greenspace. However, their strong and well-documented direct impacts on health, as well as their potential links to urban planning and greening, justify their inclusion to reduce omitted variable bias and ensure that the estimated associations are not confounded by these obvious determinants of health. Our main objective was to compare the relative health benefits of fully public greenspaces (PGS) versus general green areas (GS), rather than to disentangle all causal pathways. This study was not able to examine the specific mechanisms underlying the PGS-health relationship. Future research could employ mediation analysis or structural equation models to explore these mechanisms, with particular attention to potential mediators such as physical activity, social interaction, and environmental exposure variables. Such investigations would also provide practical insights into how PGS design, spatial patterns, and management can be optimized to better promote health through these pathways.

### 4.5 Planning and design implications

The findings offer clear, actionable insights for urban planners and policymakers. In the context of urban renewal in China, efforts to enhance urban wellbeing should prioritize the expansion and improvement of public green spaces. Notably, increasing vegetation cover or overall green space area is less effective than increasing the proportion of green space that is publicly accessible and recreationally functional.

For counties and districts that have not reached the baseline thresholds, we recommend ensuring that PGS comprises at least 12.4% of the built-up area and that PGSRatio exceeds 36.3%. For those that meet these baselines, advancing toward the identified ideal thresholds—18% for PGS and 45% for PGSRatio—would provide greater health benefits. Urban design interventions should focus on creating high-quality public green spaces with features that support physical activity, social interaction, and psychological restoration. These include diverse and inclusive amenities, safe and aesthetically pleasing environments, and easy accessibility. Moreover, urban planning departments should maintain a geospatial inventory of PGS to guide equitable allocation, prioritize underserved areas, and support ongoing monitoring and evaluation. Ultimately, public green space should be recognized not just as a recreational asset, but as a vital component of the urban health infrastructure.

## 5 Conclusion

This study provides robust empirical evidence that not all forms of urban greening contribute equally to public health. By comparing green cover (GC), general green space (GS), and public recreational green space (PGS) using a large-scale dataset of Chinese urban residents, we found that only PGS and its proportion relative to total green space (PGSRatio) were consistently associated with improved physical and mental health outcomes. These findings underscore that vegetation alone is insufficient; instead, the accessibility and functionality of green space play a critical role. Furthermore, we identified distinct nonlinear relationships between greening indicators and health. Health benefits of PGS and PGSRatio were limited below certain thresholds but became markedly positive once baseline and ideal levels—approximately 12.4–18% for PGS and 36.3–45% for PGSRatio—were surpassed. These patterns were consistent across both physical and mental health dimensions, highlighting the potential for concurrent gains through targeted urban greening interventions. Overall, the results support a behavioral pathway as the dominant mechanism through which urban green spaces improve well-being. They provide actionable insights for urban planners and public health officials aiming to design healthier, more equitable cities by emphasizing the provision of high-quality, accessible public green space.

## Data Availability

The data analyzed in this study is subject to the following licenses/restrictions: The individual-level questionnaire data from CFPS are publicly accessible at https://www.isss.pku.edu.cn/cfps. County-level data can be obtained upon reasonable request. However, the residing county information used for data matching is restricted by CFPS and is only accessible through application process within the secure server laboratory of the Institute of Social Science Survey at Peking University, Beijing, Chin. Requests to access these datasets should be directed to https://www.isss.pku.edu.cn/cfps; isss.cfps@pku.edu.cn.

## Footnotes

^1^ https://ladsweb.modaps.eosdis.nasa.gov

^2^ http://srtm.csi.cgiar.org

^3^ https://weijing-rs.github.io/product.html

## References

[1] Todd JE. Frederick law Olmstead. Boston: Twayne (1982).

[2] Bi Y Wang Y Yang D Mao J Wei Q. Urban green spaces and resident health: an empirical analysis from data across 30 provinces in China. Front Public Health. (2024) 12:1425338. doi: 10.3389/fpubh.2024.1425338, PMID: 38873324 PMC11170103

[3] Huang B Yao Z Pearce JR Feng Z James Browne A Pan Z. Non-linear association between residential greenness and general health among old adults in China. Landsc Urban Plann. (2022) 223:104406. doi: 10.1016/j.landurbplan.2022.104406

[4] Mears M Brindley P Jorgensen A Maheswaran R. Population-level linkages between urban greenspace and health inequality: the case for using multiple indicators of neighbourhood greenspace. Health Place. (2020) 62:102284. doi: 10.1016/j.healthplace.2020.102284, PMID: 32479362

[5] Zhang L Tan PY Richards D. Relative importance of quantitative and qualitative aspects of urban green spaces in promoting health. Landsc Urban Plan. (2021) 213:104131. doi: 10.1016/j.landurbplan.2021.104131

[6] Zhu W Wang J Qin B. The relationship between urban greenness and mental health: a national-level study of China. Landsc Urban Plann. (2023) 238:104830. doi: 10.1016/j.landurbplan.2023.104830

[7] Ha J Kim HJ With KA. Urban green space alone is not enough: a landscape analysis linking the spatial distribution of urban green space to mental health in the city of Chicago. Landsc Urban Plann. (2022) 218:104309. doi: 10.1016/j.landurbplan.2021.104309

[8] Jaafari S Shabani AA Moeinaddini M Danehkar A Sakieh Y. Applying landscape metrics and structural equation modeling to predict the effect of urban green space on air pollution and respiratory mortality in Tehran. Environ Monit Assess. (2020) 192:412. doi: 10.1007/s10661-020-08377-0, PMID: 32495152

[9] Nguyen PY Astell-Burt T Rahimi-Ardabili H Feng X. Green space quality and health: a systematic review. Int J Environ Res Public Health. (2021) 18:11028. doi: 10.3390/ijerph182111028, PMID: 34769549 PMC8582763

[10] Xu J Ma J Tao S. Examining the nonlinear relationship between neighborhood environment and residents' health. Cities. (2024) 152:105213. doi: 10.1016/j.cities.2024.105213

[11] Zhu W Wang J Qin B. Quantity or quality? Exploring the association between public open space and mental health in urban China. Landsc Urban Plan. (2021) 213:104128. doi: 10.1016/j.landurbplan.2021.104128

[12] Gascon M Triguero-Mas M Martínez D Dadvand P Forns J Plasència A. Mental health benefits of long-term exposure to residential green and blue spaces: a systematic review. Int J Environ Res Public Health. (2015) 12:4354–79. doi: 10.3390/ijerph120404354, PMID: 25913182 PMC4410252

[13] Song J Lu Y Zhao Q Zhang Y Yang X Chen Q. Effect modifications of green space and blue space on heat–mortality association in Hong Kong, 2008–2017. Sci Total Environ. (2022) 838:156127. doi: 10.1016/j.scitotenv.2022.156127, PMID: 35605868

[14] Fu J Fu H Zhu C Sun Y Cao H. Assessing the health risk impacts of urban green spaces on air pollution - evidence from 31 China's provinces. Ecol Indic. (2024) 159:111725. doi: 10.1016/j.ecolind.2024.111725

[15] Tomita A Vandormael AM Cuadros D Di Minin E Heikinheimo V Tanser F. Green environment and incident depression in South Africa: a geospatial analysis and mental health implications in a resource-limited setting. Lancet Planet Health. (2017) 1:e152-e162. doi: 10.1016/S2542-5196(17)30063-3, PMID: 28890948 PMC5589195

[16] Zhang J Yu Z Zhao B. Impact mechanism of urban green spaces in promoting public health: theoretical framework and inspiration for practical experiences. Landsc Archit Front. (2020) 8:104–13. doi: 10.15302/J-LAF-1-030019

[17] Zhang J Liu Y Zhou S Cheng Y Zhao B. Do various dimensions of exposure metrics affect biopsychosocial pathways linking green spaces to mental health? A cross-sectional study in Nanjing, China. Landsc Urban Plann. (2022) 226:104494. doi: 10.1016/j.landurbplan.2022.104494

[18] Cao W Zhou W Yu W Wu T. Combined effects of urban forests on land surface temperature and PM2.5 pollution in the winter and summer. Sustain Cities Soc. (2024) 104:105309. doi: 10.1016/j.scs.2024.105309

[19] Cheng X Liu J Liu H Lu S. A systematic review of evidence of additional health benefits from forest exposure. Landsc Urban Plann. (2021) 212:104123. doi: 10.1016/j.landurbplan.2021.104123

[20] Elsadek M Deshun Z Liu B. High-rise window views: evaluating the physiological and psychological impacts of green, blue, and built environments. Build Environ. (2024) 262:111798. doi: 10.1016/j.buildenv.2024.111798

[21] Ulrich RS Simons RF Losito BD Fiorito E Miles MA Zelson M. Stress recovery during exposure to natural and urban environments. J Environ Psychol. (1991) 11:201–30. doi: 10.1016/S0272-4944(05)80184-7

[22] Grigsby-Toussaint DS Turi KN Krupa M Williams NJ Pandi-Perumal SR Jean-Louis G. Sleep insufficiency and the natural environment: results from the US behavioral risk factor surveillance system survey. Prev Med. (2015) 78:78–84. doi: 10.1016/j.ypmed.2015.07.011, PMID: 26193624 PMC4818157

[23] Dadvand P Bartoll X Basagaña X Dalmau-Bueno A Martinez D Ambros A. Green spaces and general health: roles of mental health status, social support, and physical activity. Environ Int. (2016) 91:161–7. doi: 10.1016/j.envint.2016.02.029, PMID: 26949869

[24] James P Banay RF Hart JE Laden F. A review of the health benefits of greenness. Curr Epidemiol Rep. (2015) 2:131–42. doi: 10.1007/s40471-015-0043-7, PMID: 26185745 PMC4500194

[25] Liu X-X Ma X-L Huang W-Z Luo Y-N He C-J Zhong X-M. Green space and cardiovascular disease: a systematic review with meta-analysis. Environ Pollut. (2022) 301:118990. doi: 10.1016/j.envpol.2022.118990, PMID: 35181451

[26] Sun P Song Y Lu W. Effect of urban green space in the hilly environment on physical activity and health outcomes: mediation analysis on multiple greenery measures. Land. (2022) 11:612. doi: 10.3390/land11050612

[27] Hartig T Mitchell R de Vries S Frumkin H. Nature and health. Annu Rev Public Health. (2014) 35:207–28. doi: 10.1146/annurev-publhealth-032013-182443, PMID: 24387090

[28] Thompson Coon J Boddy K Stein K Whear R Barton J Depledge MH. Does participating in physical activity in outdoor natural environments have a greater effect on physical and mental wellbeing than physical activity indoors? A systematic review. Environ Sci Technol. (2011) 45:1761–72. doi: 10.1021/es102947t, PMID: 21291246

[29] Aram F Solgi E Holden G. The role of green spaces in increasing social interactions in neighborhoods with periodic markets. Habitat Int. (2019) 84:24–32. doi: 10.1016/j.habitatint.2018.12.004

[30] Cattell V Dines N Gesler W Curtis S. Mingling, observing, and lingering: everyday public spaces and their implications for well-being and social relations. Health Place. (2008) 14:544–61. doi: 10.1016/j.healthplace.2007.10.007, PMID: 18083621

[31] Liu Y Zhang F Liu Y Li Z Wu F. The effect of neighbourhood social ties on migrants' subjective wellbeing in Chinese cities. Habitat Int. (2017) 66:86–94. doi: 10.1016/j.habitatint.2017.05.011

[32] Ng TKS Gan DRY Mahendran R Kua EH Ho RC. Social connectedness as a mediator for horticultural therapy's biological effect on community-dwelling older adults: secondary analyses of a randomized controlled trial. Soc Sci Med. (2021) 284:114191. doi: 10.1016/j.socscimed.2021.114191 34271401

[33] Smith G Cirach M Swart W Dėdelė A Gidlow C van Kempen E. Characterisation of the natural environment: quantitative indicators across Europe. Int J Health Geogr. (2017) 16:16. doi: 10.1186/s12942-017-0090-z, PMID: 28446187 PMC5406880

[34] Cole HVS Triguero-Mas M Connolly JJT Anguelovski I. Determining the health benefits of green space: does gentrification matter? Health Place. (2019) 57:1–11. doi: 10.1016/j.healthplace.2019.02.001, PMID: 30844594

[35] Markevych I Schoierer J Hartig T Chudnovsky A Hystad P Dzhambov AM. Exploring pathways linking greenspace to health: theoretical and methodological guidance. Environ Res. (2017) 158:301–17. doi: 10.1016/j.envres.2017.06.028, PMID: 28672128

[36] Fan J Guo Y Cao Z Cong S Wang N Lin H. Neighborhood greenness associated with chronic obstructive pulmonary disease: a nationwide cross-sectional study in China. Environ Int. (2020) 144:106042. doi: 10.1016/j.envint.2020.106042, PMID: 32827808

[37] Sarkar C Zhang B Ni M Kumari S Bauermeister S Gallacher J. Environmental correlates of chronic obstructive pulmonary disease in 96 779 participants from the UK biobank: a cross-sectional, observational study. The Lancet Planetary Health. (2019) 3:e478–90. doi: 10.1016/S2542-5196(19)30214-1, PMID: 31777339

[38] Ji JS Zhu A Bai C Wu C-D Yan L Tang S. Residential greenness and mortality in oldest-old women and men in China: a longitudinal cohort study. Lancet Planet Health. (2019) 3:e17–25. doi: 10.1016/S2542-5196(18)30264-X, PMID: 30654864 PMC6358124

[39] Berman MG Stier AJ Akcelik GN. Environmental neuroscience. Am Psychol. (2019) 74:1039–52. doi: 10.1037/amp0000583, PMID: 31829683

[40] Slavich GM Irwin MR. From stress to inflammation and major depressive disorder: a social signal transduction theory of depression. Psychol Bull. (2014) 140:774–815. doi: 10.1037/a0035302, PMID: 24417575 PMC4006295

[41] Lian Y Li W Huang B. The impact of children migration on the health and life satisfaction of parent left behind. China Econ Q. (2014) 14:185–202. doi: 10.13821/j.cnki.ceq.2015.01.011

[42] Qi L Li Z. The Income-related Mobility of Health and Health Care Utilization. Econ Res J. (2011) 46:83–95.

[43] Yang W Kanavos P. The less healthy urban population: income-related health inequality in China. BMC Public Health. (2012) 12:804. doi: 10.1186/1471-2458-12-804, PMID: 22989200 PMC3563496

[44] Radloff LS. The CES-D scale: a self-report depression scale for research in the general population. Appl Psychol Meas. (1977) 1:385–401. doi: 10.1177/014662167700100306

[45] Wang J Wu X. The effect of intergenerational family relationships on mental health of young and middle-aged adults: gender differences and age patterns. Popul Dev. (2022) 28:98–108.

[46] Rose A McKee J Sims K Bright E Reith A Urban M. LandScan Global 2020. Oak Ridge, TN: Oak Ridge National Laboratory (2021).

[47] Jarvis A Reuter HI Nelson A Guevara E. Hole-filled seamless SRTM data V4 International Centre for Tropical Agriculture (CIAT) (2008).

[48] Peng S. 1-km monthly mean temperature dataset for China (1901–2023) National Tibetan Plateau Data Center (2024).

[49] Zhang H Luo M Zhan W Zhao Y. A first 1 km high-resolution atmospheric moisture index collection over China, 2003–2020 National Tibetan Plateau Data Center (2023). 10.1038/s41597-024-03230-2 PMC11043353 38658632

[50] Wei J Li Z Lyapustin A Sun L Peng Y Xue W. Reconstructing 1-km-resolution high-quality PM2.5 data records from 2000 to 2018 in China: spatiotemporal variations and policy implications. Remote Sens Environ. (2021) 252:112136. doi: 10.1016/j.rse.2020.112136

[51] Chen T. Guestrin C. (2016). XGBoost: a scalable tree boosting system. Proceedings of the 22nd ACM SIGKDD International Conference on Knowledge Discovery and Data Mining 785–794. San Francisco, CA: Association for Computing Machinery.

[52] Zamani Joharestani M Cao C Ni X Bashir B Talebiesfandarani S. PM2.5 prediction based on random Forest, XGBoost, and deep learning using multisource remote sensing Data. Atmos. (2019) 10:373. doi: 10.3390/atmos10070373

[53] Chen Y Zhang X Grekousis G Huang Y Hua F Pan Z. Examining the importance of built and natural environment factors in predicting self-rated health in older adults: an extreme gradient boosting (XGBoost) approach. J Clean Prod. (2023) 413:137432. doi: 10.1016/j.jclepro.2023.137432

[54] Liu Z Liu C. The association between urban density and multiple health risks based on interpretable machine learning: a study of American urban communities. Cities. (2024) 153:105170. doi: 10.1016/j.cities.2024.105170

[55] Lundberg S. M. Lee S.-I. (2017). A unified approach to interpreting model predictions. Proceedings of the 31st International Conference on Neural Information Processing Systems. Long Beach, California, USA: Curran Associates Inc. 4765–4774. doi: 10.5555/3295222.3295230

[56] Veitch J Timperio A Salmon J Hall SJ Abbott G Flowers EP. Examination of the acute heart rate and salivary cortisol response to a single bout of walking in urban and green environments: a pilot study. Urban For Urban Green. (2022) 74:127660. doi: 10.1016/j.ufug.2022.127660

[57] Wang H Dai X Wu J Wu X Nie X. Influence of urban green open space on residents’ physical activity in China. BMC Public Health. (2019) 19:1093. doi: 10.1186/s12889-019-7416-7, PMID: 31409316 PMC6693084

[58] Ekkel ED de Vries S. Nearby green space and human health: evaluating accessibility metrics. Landsc Urban Plann. (2017) 157:214–20. doi: 10.1016/j.landurbplan.2016.06.008

[59] van den Berg M Wendel-Vos W van Poppel M Kemper H van Mechelen W Maas J. Health benefits of green spaces in the living environment: a systematic review of epidemiological studies. Urban For Urban Green. (2015) 14:806–16. doi: 10.1016/j.ufug.2015.07.008

[60] Kaplan S. The restorative benefits of nature: toward an integrative framework. J Environ Psychol. (1995) 15:169–82. doi: 10.1016/0272-4944(95)90001-2

[61] Woodridge JM. Econometric analysis of cross section and panel Data. 2nd ed. Cambridge: MIT Press (2010).

[62] UN-Habitat. Urbanization and development: emerging futures. Nairobi: United Nations Human Settlements Programme (2016).

[63] Zhang L Tan PY Gan DRY Samsudin R. Assessment of mediators in the associations between urban green spaces and self-reported health. Landsc Urban Plann. (2022) 226:104503. doi: 10.1016/j.landurbplan.2022.104503

