# Supplementary material for: Nonlinear health benefits of public green space: evidence from a nationwide machine learning study in China
Source: Front Public Health. 2025 Oct 28;13:1680591. doi: 10.3389/fpubh.2025.1680591 (PMC12602450; doi:10.3389/fpubh.2025.1680591)
Supplement: Supplementary file 1 [file Data_Sheet_1.docx]

**Appendix I**

**Table S1** Description of the variables.

| Variable | Definition |  | Scale/Score |
| --- | --- | --- | --- |
| ***Dependent variable*** |  |  |  |
| Health_physical5* | How would you rate your health status? |  | 1. Poor 2. Fair 3. Good  4. Very good 5. Excellent |
| Health_physical |  |  | 1. Good physical health  0. Poor physical health |
| Health_mental | Respondents' total score derived from eight question items describing their mental status. | The CES-D scale:  During the past week,  ① I felt depressed.  ② I felt that everything I did was an effort.  ③ My sleep was restless.  ④ I was happy.  ⑤ I felt lonely.  ⑥ I enjoyed life.  ⑦ I felt sad.  ⑧ I could not get "going." | 1. Most of the time (5-7 days)  2. Often (3-4 days)  3. Sometimes (1-2 days)  4. Never (less than one day) |
| Health_mental01* |  |  | 1. Good mental health  0. Poor mental health |
| Health_hospitalized* | In the past year, were you ever been hospitalized due to illness? |  | 1. Yes  0. No |
| ***Independent variable*** |  |  |  |
| GC | Proportion of green cover within urban built-up area |  | 0 - 100 |
| GS | Proportion of general green space within urban built-up area | Include four major categories: public recreational green spaces, green buffers, squares, and attached green spaces. | 0 - 100 |
| PGS | Proportion of public recreational green space within urban built-up area | Include four major categories: urban parks, community parks, special use parks, and mini parks. | 0 - 100 |
| PGSRatio | Ratio of public recreational green space to green space | GS/PGS | 0 - 100 |
| ***Control variable - individual level*** | | | |
| Age |  |  |  |
| Gender |  |  | 1. Male 0. Female |
| Education | Years of education completed by individual questionnaire respondent |  | 0-22 years |
| Marriage | Marital status of the respondents |  | 1. Married or cohabitation  0. Never married, divorced or widowed |
| Income | What is your relative income level in your local area? |  | 1. Very low  2. Low 3. Medium  4. High 5. Very high |
| Smoke | Did you smoke cigarettes in the past month? |  | 1. Yes  0. No |
| Drink | Did you drink alcohol at least 3 times a week in the past month? |  | 1. Yes  0. No |
| Exercise | In the past 12 months, how often did you participate in physical fitness and leisure activities (Not including cycling or walking for the single purpose of commuting)? |  | 0. Never  1. Less than once a month  2. More than once a month, but less than once a week  3. One to two times a week  4. three to four times a week  5. More than five times a week  6. Once a day  7. Twice a day or more |
| Overweight | Does the respondent have a body mass index (BMI) of 28 or above? |  | 1. Yes  0. No |
| Underweight | Does the respondent have a BMI of 18.5 or below? |  | 1. Yes  0. No |
| ***Control variable - county level*** | | | |
| lnGDP | The logarithm of gross domestic product (GDP) |  |  |
| PropofIndustry | Share of secondary sector in GDP (%) |  | 0 - 1 |
| lnDisposableIncome | The logarithm of the per capita disposable income of urban residents |  |  |
| lnPopDens | Population per square kilometer |  |  |
| DEM | Digital elevation model (m) |  |  |
| RH | Annual average relative humidity (%) |  | 0 - 1 |
| TempAve | Annual average air temperature (℃) |  |  |
| PM_2.5_ | Annual average PM_2.5_ concentration (μg/m^3^) |  |  |
| SunshineHours* | Annual sunshine hours |  |  |

Note: *Variables were used in the robustness tests.

**Table S2** Spearman correlation of personal characteristics vs SRH.

|  | Health  _physical | Health  _mental | Age | Gender | Education | Marriage | Income | Smoke | Drink | Exercise | Over-  weight | Under-  weight |
| --- | --- | --- | --- | --- | --- | --- | --- | --- | --- | --- | --- | --- |
| Health  _physical | 1 |  |  |  |  |  |  |  |  |  |  |  |
| Health  _mental | 0.209* | 1 |  |  |  |  |  |  |  |  |  |  |
| Age | -0.290* | 0.064* | 1 |  |  |  |  |  |  |  |  |  |
| Gender | 0.074* | 0.101* | 0.011 | 1 |  |  |  |  |  |  |  |  |
| Education | 0.245* | 0.070* | -0.503* | 0.074* | 1 |  |  |  |  |  |  |  |
| Marriage | -0.041* | 0.084* | 0.250* | 0.002 | -0.123* | 1 |  |  |  |  |  |  |
| Income | 0.104* | 0.168* | 0.127* | 0.034* | -0.055* | 0.072* | 1 |  |  |  |  |  |
| Smoke | 0.058* | 0.024* | -0.002 | 0.564* | -0.012 | 0.016 | -0.0004 | 1 |  |  |  |  |
| Drink | 0.040* | 0.045* | 0.088* | 0.331* | -0.052* | 0.054* | 0.034* | 0.303* | 1 |  |  |  |
| Exercise | 0.043* | 0.096* | 0.077* | 0.046* | 0.205* | -0.063* | 0.028* | -0.032* | -0.011 | 1 |  |  |
| Overweight | -0.057* | 0.024* | 0.008 | 0.073* | 0.005 | 0.034* | 0.013 | 0.042* | 0.043* | 0.013 | 1 |  |
| Underweight | -0.014 | -0.031* | -0.120* | -0.087* | 0.033* | -0.146* | -0.024* | -0.033* | -0.036* | -0.029* | -0.082* | 1 |

Note: * p < 0.05.

**Table S3** Spearman correlation of county-level characteristics vs SRH.

|  | Health  _physical | Health  _mental | lnGDP | Propof  Industry | lnDisposable  Income | lnPopDens | DEM | RH | Temp  Ave | PM_2.5_ | NDVI |
| --- | --- | --- | --- | --- | --- | --- | --- | --- | --- | --- | --- |
| Health  _physical | 1 |  |  |  |  |  |  |  |  |  |  |
| Health  _mental | 0.209* | 1 |  |  |  |  |  |  |  |  |  |
| lnGDP | -0.028* | 0.109* | 1 |  |  |  |  |  |  |  |  |
| Propof  Industry | 0.003 | 0.026* | 0.391* | 1 |  |  |  |  |  |  |  |
| lnDisposable  Income | -0.017 | 0.107* | 0.794* | 0.284* | 1 |  |  |  |  |  |  |
| lnPopDens | -0.019 | 0.097* | 0.715* | 0.158* | 0.629* | 1 |  |  |  |  |  |
| DEM | 0.036* | -0.100* | -0.661* | -0.241* | -0.539* | -0.669* | 1 |  |  |  |  |
| RH | -0.063* | 0.006 | 0.282* | 0.191* | 0.196* | 0.016 | -0.278* | 1 |  |  |  |
| TempAve | -0.054* | 0.022* | 0.513* | 0.276* | 0.402* | 0.328* | -0.491* | 0.754* | 1 |  |  |
| PM_2.5_ | 0.046* | 0.031* | 0.025* | 0.066* | -0.030* | 0.248* | -0.158* | -0.502* | -0.291* | 1 |  |
| NDVI | -0.038* | -0.064* | -0.268* | 0.025* | -0.313* | -0.488* | 0.254* | 0.619* | 0.298* | -0.279* | 1 |

Note: * p < 0.05.

**Table S4** Spearman correlation of GS vs county-level characteristics.

|  | GC | GS | PGS | PGSRatio | lnGDP | Propof  Industry | lnDisposable  Income | lnPopDens | DEM | RH | Temp-Ave | PM_2.5_ | NDVI |
| --- | --- | --- | --- | --- | --- | --- | --- | --- | --- | --- | --- | --- | --- |
| GC | 1 |  |  |  |  |  |  |  |  |  |  |  |  |
| GS | 0.897* | 1 |  |  |  |  |  |  |  |  |  |  |  |
| PGS | 0.464* | 0.445* | 1 |  |  |  |  |  |  |  |  |  |  |
| PGSRatio | 0.020 | -0.031* | 0.782* | 1 |  |  |  |  |  |  |  |  |  |
| lnGDP | 0.460* | 0.417* | 0.147* | 0.104* | 1 |  |  |  |  |  |  |  |  |
| Propof  Industry | 0.334* | 0.335* | 0.024* | -0.064* | 0.391* | 1 |  |  |  |  |  |  |  |
| lnDisposable  Income | 0.370* | 0.320* | 0.132* | 0.125* | 0.794* | 0.284* | 1 |  |  |  |  |  |  |
| lnPopDens | 0.431* | 0.393* | 0.129* | 0.016 | 0.715* | 0.158* | 0.629* | 1 |  |  |  |  |  |
| DEM | -0.371* | -0.399* | -0.025* | 0.032* | -0.661* | -0.241* | -0.539* | -0.669* | 1 |  |  |  |  |
| RH | 0.178* | 0.182* | 0.139* | 0.140* | 0.282* | 0.191* | 0.196* | 0.016 | -0.278* | 1 |  |  |  |
| TempAve | 0.334* | 0.289* | 0.182* | 0.134* | 0.513* | 0.276* | 0.402* | 0.328* | -0.491* | 0.754* | 1 |  |  |
| PM_2.5_ | 0.078* | 0.004 | -0.139* | -0.266* | 0.025* | 0.066* | -0.030* | 0.248* | -0.158* | -0.502* | -0.291* | 1 |  |
| NDVI | -0.055* | -0.042* | 0.024* | -0.052* | -0.268* | 0.025* | -0.313* | -0.488* | 0.254* | 0.619* | 0.298* | -0.279* | 1 |

Note: * p < 0.05.

**Table S5** Regression analysis of the relationship between urban greening and physical and mental health (baseline).

| Model | Health_physical | | | |  | Health_mental | | | |  |
| --- | --- | --- | --- | --- | --- | --- | --- | --- | --- | --- |
|  | (a) | (b) | (c) | (d) |  | (e) | (f) | (g) | (h) |  |
| **GC** | **0.004** |  |  |  |  | **0.008** |  |  |  |  |
|  | (0.005) |  |  |  |  | (0.008) |  |  |  |  |
| **GS** |  | **0.006** |  |  |  |  | **0.008** |  |  |  |
|  |  | (0.005) |  |  |  |  | (0.007) |  |  |  |
| **PGS** |  |  | **0.025^***^** |  |  |  |  | **0.021^**^** |  |  |
|  |  |  | (0.006) |  |  |  |  | (0.009) |  |  |
| **PGSRatio** |  |  |  | **0.006^**^** |  |  |  |  | **0.008^**^** |  |
|  |  |  |  | (0.002) |  |  |  |  | (0.003) |  |
| Age | -0.045^***^ | -0.045^***^ | -0.044^***^ | -0.045^***^ |  | 0.004 | 0.002 | 0.005 | 0.002 |  |
|  | (0.002) | (0.002) | (0.002) | (0.002) |  | (0.003) | (0.004) | (0.003) | (0.004) |  |
| 0.Gender | 0.000 | 0.000 | 0.000 | 0.000 |  | 0.000 | 0.000 | 0.000 | 0.000 |  |
|  | (.) | (.) | (.) | (.) |  | (.) | (.) | (.) | (.) |  |
| 1.Gender | 0.202^***^ | 0.193^***^ | 0.196^***^ | 0.195^***^ |  | 0.665^***^ | 0.685^***^ | 0.702^***^ | 0.686^***^ |  |
|  | (0.071) | (0.072) | (0.069) | (0.072) |  | (0.103) | (0.105) | (0.101) | (0.105) |  |
| Education | 0.065^***^ | 0.066^***^ | 0.066^***^ | 0.065^***^ |  | 0.083^***^ | 0.079^***^ | 0.079^***^ | 0.079^***^ |  |
|  | (0.007) | (0.007) | (0.007) | (0.007) |  | (0.012) | (0.012) | (0.011) | (0.012) |  |
| 0.Marriage | 0.000 | 0.000 | 0.000 | 0.000 |  | 0.000 | 0.000 | 0.000 | 0.000 |  |
|  | (.) | (.) | (.) | (.) |  | (.) | (.) | (.) | (.) |  |
| 1.Marriage | -0.038 | -0.033 | -0.042 | -0.035 |  | 0.864^***^ | 0.855^***^ | 0.876^***^ | 0.852^***^ |  |
|  | (0.082) | (0.083) | (0.080) | (0.083) |  | (0.120) | (0.122) | (0.118) | (0.122) |  |
| Income | 0.363^***^ | 0.366^***^ | 0.365^***^ | 0.366^***^ |  | 0.657^***^ | 0.650^***^ | 0.650^***^ | 0.648^***^ |  |
|  | (0.028) | (0.028) | (0.027) | (0.028) |  | (0.046) | (0.047) | (0.045) | (0.047) |  |
| 0.Smoke | 0.000 | 0.000 | 0.000 | 0.000 |  | 0.000 | 0.000 | 0.000 | 0.000 |  |
|  | (.) | (.) | (.) | (.) |  | (.) | (.) | (.) | (.) |  |
| 1.Smoke | 0.132 | 0.130 | 0.123 | 0.123 |  | -0.317^***^ | -0.339^***^ | -0.313^***^ | -0.347^***^ |  |
|  | (0.081) | (0.082) | (0.079) | (0.083) |  | (0.116) | (0.118) | (0.113) | (0.118) |  |
| 0.Drink | 0.000 | 0.000 | 0.000 | 0.000 |  | 0.000 | 0.000 | 0.000 | 0.000 |  |
|  | (.) | (.) | (.) | (.) |  | (.) | (.) | (.) | (.) |  |
| 1.Drink | 0.302^***^ | 0.331^***^ | 0.357^***^ | 0.342^***^ |  | 0.083 | 0.091 | 0.111 | 0.104 |  |
|  | (0.093) | (0.096) | (0.091) | (0.096) |  | (0.129) | (0.133) | (0.126) | (0.133) |  |
| Exercise | 0.041^***^ | 0.036^***^ | 0.040^***^ | 0.036^***^ |  | 0.103^***^ | 0.100^***^ | 0.103^***^ | 0.101^***^ |  |
|  | (0.012) | (0.012) | (0.011) | (0.012) |  | (0.018) | (0.018) | (0.017) | (0.018) |  |
| 0.Overweight | 0.000 | 0.000 | 0.000 | 0.000 |  | 0.000 | 0.000 | 0.000 | 0.000 |  |
|  | (.) | (.) | (.) | (.) |  | (.) | (.) | (.) | (.) |  |
| 1.Overweight | -0.601^***^ | -0.617^***^ | -0.612^***^ | -0.615^***^ |  | 0.035 | 0.017 | 0.037 | 0.020 |  |
|  | (0.091) | (0.093) | (0.088) | (0.093) |  | (0.144) | (0.147) | (0.139) | (0.147) |  |
| 0.Underweight | 0.000 | 0.000 | 0.000 | 0.000 |  | 0.000 | 0.000 | 0.000 | 0.000 |  |
|  | (.) | (.) | (.) | (.) |  | (.) | (.) | (.) | (.) |  |
| 1.Underweight | -0.421^***^ | -0.410^***^ | -0.407^***^ | -0.421^***^ |  | 0.004 | -0.003 | 0.036 | -0.016 |  |
|  | (0.121) | (0.123) | (0.119) | (0.123) |  | (0.181) | (0.183) | (0.177) | (0.183) |  |
| lnGDP | -0.078^*^ | -0.068 | -0.060 | -0.060 |  | 0.067 | 0.088 | 0.054 | 0.100 |  |
|  | (0.043) | (0.047) | (0.042) | (0.047) |  | (0.062) | (0.067) | (0.061) | (0.067) |  |
| PropofIndustry | 0.127 | 0.134 | 0.135 | 0.232 |  | -0.248 | -0.321 | 0.004 | -0.191 |  |
|  | (0.212) | (0.221) | (0.197) | (0.217) |  | (0.315) | (0.328) | (0.295) | (0.320) |  |
| lnDisposableIncome | 0.101 | 0.105 | 0.017 | 0.066 |  | 0.683^***^ | 0.641^***^ | 0.829^***^ | 0.601^**^ |  |
|  | (0.161) | (0.162) | (0.155) | (0.162) |  | (0.244) | (0.245) | (0.238) | (0.245) |  |
| lnPopDens | -0.042 | -0.044 | -0.049 | -0.037 |  | -0.044 | -0.048 | -0.039 | -0.040 |  |
|  | (0.032) | (0.033) | (0.032) | (0.032) |  | (0.047) | (0.047) | (0.046) | (0.047) |  |
| DEM | 0.000 | 0.000 | 0.000 | 0.000 |  | -0.000 | -0.000 | -0.000 | -0.000^*^ |  |
|  | (0.000) | (0.000) | (0.000) | (0.000) |  | (0.000) | (0.000) | (0.000) | (0.000) |  |
| RH | 1.062 | 1.145 | 1.146 | 1.111 |  | 2.836^**^ | 2.609^**^ | 3.447^***^ | 2.528^**^ |  |
|  | (0.791) | (0.797) | (0.783) | (0.798) |  | (1.223) | (1.234) | (1.215) | (1.229) |  |
| TempAve | -0.011 | -0.012 | -0.016^*^ | -0.016^*^ |  | -0.041^***^ | -0.041^***^ | -0.051^***^ | -0.047^***^ |  |
|  | (0.009) | (0.009) | (0.009) | (0.009) |  | (0.013) | (0.013) | (0.013) | (0.014) |  |
| PM_2.5_ | 0.011^***^ | 0.011^***^ | 0.014^***^ | 0.012^***^ |  | 0.012^**^ | 0.012^**^ | 0.015^**^ | 0.014^**^ |  |
|  | (0.004) | (0.004) | (0.004) | (0.004) |  | (0.006) | (0.006) | (0.006) | (0.006) |  |
| NDVI | -1.605^***^ | -1.634^***^ | -1.513^***^ | -1.460^***^ |  | -0.890 | -0.767 | -0.935 | -0.511 |  |
|  | (0.460) | (0.461) | (0.455) | (0.463) |  | (0.689) | (0.690) | (0.676) | (0.683) |  |
| _cons | 1.629 | 1.348 | 1.982 | 1.552 |  | 13.185^***^ | 13.653^***^ | 11.422^***^ | 13.776^***^ |  |
|  | (1.452) | (1.482) | (1.393) | (1.463) |  | (2.257) | (2.305) | (2.193) | (2.274) |  |
| *N* | 8538 | 8272 | 8897 | 8272 |  | 8503 | 8238 | 8860 | 8238 |  |
| Pseudo/adj. *R*^2^ | 0.132 | 0.131 | 0.133 | 0.132 |  | 0.068 | 0.064 | 0.072 | 0.064 |  |

Note: Standard errors clustered at the county/district level are in parentheses. *** p < 0.01, ** p < 0.05, * p < 0.1.

**Table S6** Regression analysis of the relationship between urban greening and physical and mental health (Robustness test 1).

| Model | Health_physical | | | |  | Health_mental | | | |  |
| --- | --- | --- | --- | --- | --- | --- | --- | --- | --- | --- |
|  | (a_R1) | (b_R1) | (c_R1) | (d_R1) |  | (e_R1) | (f_R1) | (g_R1) | (h_R1) |  |
| **GC** | **0.005** |  |  |  |  | **0.008** |  |  |  |  |
|  | (0.005) |  |  |  |  | (0.008) |  |  |  |  |
| **GS** |  | **0.006** |  |  |  |  | **0.005** |  |  |  |
|  |  | (0.005) |  |  |  |  | (0.008) |  |  |  |
| **PGS** |  |  | **0.027^***^** |  |  |  |  | **0.021^**^** |  |  |
|  |  |  | (0.007) |  |  |  |  | (0.011) |  |  |
| **PGSRatio** |  |  |  | **0.006^**^** |  |  |  |  | **0.009^**^** |  |
|  |  |  |  | (0.003) |  |  |  |  | (0.004) |  |
| Age | -0.045^***^ | -0.045^***^ | -0.044^***^ | -0.045^***^ |  | 0.004 | 0.002 | 0.005 | 0.002 |  |
|  | (0.002) | (0.002) | (0.002) | (0.002) |  | (0.003) | (0.004) | (0.003) | (0.004) |  |
| 0.Gender | 0.000 | 0.000 | 0.000 | 0.000 |  | 0.000 | 0.000 | 0.000 | 0.000 |  |
|  | (.) | (.) | (.) | (.) |  | (.) | (.) | (.) | (.) |  |
| 1.Gender | 0.201^***^ | 0.193^***^ | 0.196^***^ | 0.194^***^ |  | 0.661^***^ | 0.682^***^ | 0.698^***^ | 0.682^***^ |  |
|  | (0.071) | (0.072) | (0.069) | (0.072) |  | (0.103) | (0.105) | (0.101) | (0.105) |  |
| Education | 0.065^***^ | 0.066^***^ | 0.066^***^ | 0.065^***^ |  | 0.084^***^ | 0.080^***^ | 0.080^***^ | 0.080^***^ |  |
|  | (0.007) | (0.007) | (0.007) | (0.007) |  | (0.012) | (0.012) | (0.011) | (0.012) |  |
| 0.Marriage | 0.000 | 0.000 | 0.000 | 0.000 |  | 0.000 | 0.000 | 0.000 | 0.000 |  |
|  | (.) | (.) | (.) | (.) |  | (.) | (.) | (.) | (.) |  |
| 1.Marriage | -0.041 | -0.036 | -0.046 | -0.037 |  | 0.861^***^ | 0.852^***^ | 0.872^***^ | 0.850^***^ |  |
|  | (0.082) | (0.083) | (0.080) | (0.083) |  | (0.120) | (0.122) | (0.118) | (0.122) |  |
| Income | 0.363^***^ | 0.366^***^ | 0.365^***^ | 0.366^***^ |  | 0.656^***^ | 0.649^***^ | 0.650^***^ | 0.648^***^ |  |
|  | (0.028) | (0.028) | (0.027) | (0.028) |  | (0.046) | (0.047) | (0.045) | (0.047) |  |
| 0.Smoke | 0.000 | 0.000 | 0.000 | 0.000 |  | 0.000 | 0.000 | 0.000 | 0.000 |  |
|  | (.) | (.) | (.) | (.) |  | (.) | (.) | (.) | (.) |  |
| 1.Smoke | 0.132 | 0.129 | 0.122 | 0.124 |  | -0.314^***^ | -0.338^***^ | -0.310^***^ | -0.345^***^ |  |
|  | (0.081) | (0.082) | (0.079) | (0.083) |  | (0.116) | (0.118) | (0.113) | (0.118) |  |
| 0.Drink | 0.000 | 0.000 | 0.000 | 0.000 |  | 0.000 | 0.000 | 0.000 | 0.000 |  |
|  | (.) | (.) | (.) | (.) |  | (.) | (.) | (.) | (.) |  |
| 1.Drink | 0.300^***^ | 0.330^***^ | 0.355^***^ | 0.340^***^ |  | 0.092 | 0.100 | 0.118 | 0.113 |  |
|  | (0.093) | (0.096) | (0.091) | (0.096) |  | (0.129) | (0.133) | (0.126) | (0.133) |  |
| Exercise | 0.042^***^ | 0.037^***^ | 0.041^***^ | 0.037^***^ |  | 0.102^***^ | 0.100^***^ | 0.103^***^ | 0.101^***^ |  |
|  | (0.012) | (0.012) | (0.011) | (0.012) |  | (0.018) | (0.018) | (0.017) | (0.018) |  |
| 0.Overweight | 0.000 | 0.000 | 0.000 | 0.000 |  | 0.000 | 0.000 | 0.000 | 0.000 |  |
|  | (.) | (.) | (.) | (.) |  | (.) | (.) | (.) | (.) |  |
| 1.Overweight | -0.602^***^ | -0.618^***^ | -0.611^***^ | -0.615^***^ |  | 0.034 | 0.017 | 0.037 | 0.021 |  |
|  | (0.091) | (0.093) | (0.088) | (0.093) |  | (0.144) | (0.147) | (0.139) | (0.147) |  |
| 0.Underweight | 0.000 | 0.000 | 0.000 | 0.000 |  | 0.000 | 0.000 | 0.000 | 0.000 |  |
|  | (.) | (.) | (.) | (.) |  | (.) | (.) | (.) | (.) |  |
| 1.Underweight | -0.422^***^ | -0.412^***^ | -0.407^***^ | -0.421^***^ |  | 0.002 | -0.005 | 0.036 | -0.017 |  |
|  | (0.121) | (0.123) | (0.119) | (0.123) |  | (0.181) | (0.183) | (0.177) | (0.183) |  |
| lnGDP | -0.072 | -0.061 | -0.062 | -0.062 |  | 0.135^*^ | 0.151^**^ | 0.115^*^ | 0.148^**^ |  |
|  | (0.049) | (0.051) | (0.049) | (0.050) |  | (0.071) | (0.072) | (0.070) | (0.072) |  |
| PropofIndustry | 0.150 | 0.163 | 0.193 | 0.301 |  | -0.372 | -0.417 | -0.142 | -0.238 |  |
|  | (0.223) | (0.231) | (0.210) | (0.226) |  | (0.335) | (0.345) | (0.317) | (0.340) |  |
| lnDisposableIncome | 0.045 | 0.057 | -0.018 | 0.026 |  | 0.490^*^ | 0.454^*^ | 0.653^**^ | 0.423 |  |
|  | (0.173) | (0.175) | (0.168) | (0.175) |  | (0.260) | (0.262) | (0.254) | (0.262) |  |
| lnPopDens | -0.055 | -0.052 | -0.061^*^ | -0.039 |  | -0.042 | -0.041 | -0.033 | -0.027 |  |
|  | (0.035) | (0.035) | (0.034) | (0.035) |  | (0.051) | (0.051) | (0.050) | (0.051) |  |
| DEM | 0.000 | 0.000 | 0.000 | 0.000 |  | -0.000 | -0.000^*^ | -0.000 | -0.000^*^ |  |
|  | (0.000) | (0.000) | (0.000) | (0.000) |  | (0.000) | (0.000) | (0.000) | (0.000) |  |
| RH | 1.162 | 1.207 | 1.287 | 1.174 |  | 2.581^**^ | 2.269^*^ | 3.264^**^ | 2.219^*^ |  |
|  | (0.835) | (0.841) | (0.828) | (0.842) |  | (1.290) | (1.298) | (1.286) | (1.295) |  |
| TempAve | -0.014 | -0.015 | -0.018^**^ | -0.019^**^ |  | -0.043^***^ | -0.042^***^ | -0.051^***^ | -0.047^***^ |  |
|  | (0.009) | (0.009) | (0.009) | (0.009) |  | (0.014) | (0.014) | (0.014) | (0.014) |  |
| PM_2.5_ | 0.011^***^ | 0.011^***^ | 0.013^***^ | 0.012^***^ |  | 0.011^*^ | 0.011^*^ | 0.013^**^ | 0.013^**^ |  |
|  | (0.004) | (0.004) | (0.004) | (0.004) |  | (0.006) | (0.006) | (0.006) | (0.006) |  |
| NDVI | -1.704^***^ | -1.678^***^ | -1.651^***^ | -1.505^***^ |  | -0.735 | -0.555 | -0.815 | -0.312 |  |
|  | (0.470) | (0.469) | (0.463) | (0.472) |  | (0.710) | (0.704) | (0.696) | (0.702) |  |
| _cons | 2.218 | 1.863 | 2.453^*^ | 2.027 |  | 14.424^***^ | 14.938^***^ | 12.490^***^ | 14.968^***^ |  |
|  | (1.519) | (1.568) | (1.468) | (1.551) |  | (2.334) | (2.415) | (2.279) | (2.384) |  |
| *N* | 8538 | 8272 | 8897 | 8272 |  | 8503 | 8238 | 8860 | 8238 |  |
| Pseudo/adj. *R*^2^ | 0.132 | 0.131 | 0.133 | 0.132 |  | 0.068 | 0.064 | 0.071 | 0.064 |  |

Note: Standard errors clustered at the county/district level are in parentheses. *** p < 0.01, ** p < 0.05, * p < 0.1.

**Table S7** Regression analysis of the relationship between urban greening and physical and mental health (Robustness test 2)

| Model | Health_physical | | | |  | Health_mental | | | |  |
| --- | --- | --- | --- | --- | --- | --- | --- | --- | --- | --- |
|  | (a_R2) | (b_R2) | (c_R2) | (d_R2) |  | (e_R2) | (f_R2) | (g_R2) | (h_R2) |  |
| **GC** | **0.004** |  |  |  |  | **0.004** |  |  |  |  |
|  | (0.005) |  |  |  |  | (0.008) |  |  |  |  |
| **GS** |  | **0.005** |  |  |  |  | **0.003** |  |  |  |
|  |  | (0.005) |  |  |  |  | (0.007) |  |  |  |
| **PGS** |  |  | **0.025^***^** |  |  |  |  | **0.020^**^** |  |  |
|  |  |  | (0.006) |  |  |  |  | (0.009) |  |  |
| **PGSRatio** |  |  |  | **0.006^***^** |  |  |  |  | **0.009^***^** |  |
|  |  |  |  | (0.002) |  |  |  |  | (0.003) |  |
| Age | -0.045^***^ | -0.045^***^ | -0.044^***^ | -0.045^***^ |  | 0.004 | 0.002 | 0.006^*^ | 0.002 |  |
|  | (0.002) | (0.002) | (0.002) | (0.002) |  | (0.003) | (0.004) | (0.003) | (0.004) |  |
| 0.Gender | 0.000 | 0.000 | 0.000 | 0.000 |  | 0.000 | 0.000 | 0.000 | 0.000 |  |
|  | (.) | (.) | (.) | (.) |  | (.) | (.) | (.) | (.) |  |
| 1.Gender | 0.202^***^ | 0.194^***^ | 0.196^***^ | 0.195^***^ |  | 0.667^***^ | 0.688^***^ | 0.703^***^ | 0.688^***^ |  |
|  | (0.071) | (0.072) | (0.069) | (0.072) |  | (0.103) | (0.105) | (0.101) | (0.105) |  |
| Education | 0.065^***^ | 0.066^***^ | 0.066^***^ | 0.066^***^ |  | 0.084^***^ | 0.081^***^ | 0.080^***^ | 0.081^***^ |  |
|  | (0.007) | (0.007) | (0.007) | (0.007) |  | (0.012) | (0.012) | (0.011) | (0.012) |  |
| 0.Marriage | 0.000 | 0.000 | 0.000 | 0.000 |  | 0.000 | 0.000 | 0.000 | 0.000 |  |
|  | (.) | (.) | (.) | (.) |  | (.) | (.) | (.) | (.) |  |
| 1.Marriage | -0.038 | -0.033 | -0.042 | -0.036 |  | 0.860^***^ | 0.852^***^ | 0.873^***^ | 0.848^***^ |  |
|  | (0.082) | (0.083) | (0.080) | (0.083) |  | (0.120) | (0.122) | (0.117) | (0.122) |  |
| Income | 0.363^***^ | 0.366^***^ | 0.364^***^ | 0.365^***^ |  | 0.655^***^ | 0.648^***^ | 0.648^***^ | 0.647^***^ |  |
|  | (0.028) | (0.028) | (0.027) | (0.028) |  | (0.046) | (0.047) | (0.045) | (0.047) |  |
| 0.Smoke | 0.000 | 0.000 | 0.000 | 0.000 |  | 0.000 | 0.000 | 0.000 | 0.000 |  |
|  | (.) | (.) | (.) | (.) |  | (.) | (.) | (.) | (.) |  |
| 1.Smoke | 0.133 | 0.130 | 0.124 | 0.124 |  | -0.316^***^ | -0.340^***^ | -0.311^***^ | -0.347^***^ |  |
|  | (0.081) | (0.082) | (0.079) | (0.083) |  | (0.116) | (0.118) | (0.113) | (0.118) |  |
| 0.Drink | 0.000 | 0.000 | 0.000 | 0.000 |  | 0.000 | 0.000 | 0.000 | 0.000 |  |
|  | (.) | (.) | (.) | (.) |  | (.) | (.) | (.) | (.) |  |
| 1.Drink | 0.303^***^ | 0.331^***^ | 0.358^***^ | 0.342^***^ |  | 0.086 | 0.091 | 0.111 | 0.103 |  |
|  | (0.093) | (0.096) | (0.091) | (0.096) |  | (0.129) | (0.133) | (0.126) | (0.133) |  |
| Exercise | 0.041^***^ | 0.036^***^ | 0.040^***^ | 0.036^***^ |  | 0.103^***^ | 0.101^***^ | 0.104^***^ | 0.101^***^ |  |
|  | (0.012) | (0.012) | (0.011) | (0.012) |  | (0.018) | (0.018) | (0.017) | (0.018) |  |
| 0.Overweight | 0.000 | 0.000 | 0.000 | 0.000 |  | 0.000 | 0.000 | 0.000 | 0.000 |  |
|  | (.) | (.) | (.) | (.) |  | (.) | (.) | (.) | (.) |  |
| 1.Overweight | -0.603^***^ | -0.619^***^ | -0.614^***^ | -0.619^***^ |  | 0.024 | 0.006 | 0.027 | 0.007 |  |
|  | (0.091) | (0.093) | (0.089) | (0.093) |  | (0.144) | (0.147) | (0.139) | (0.147) |  |
| 0.Underweight | 0.000 | 0.000 | 0.000 | 0.000 |  | 0.000 | 0.000 | 0.000 | 0.000 |  |
|  | (.) | (.) | (.) | (.) |  | (.) | (.) | (.) | (.) |  |
| 1.Underweight | -0.421^***^ | -0.410^***^ | -0.406^***^ | -0.420^***^ |  | 0.011 | 0.003 | 0.045 | -0.009 |  |
|  | (0.121) | (0.123) | (0.119) | (0.123) |  | (0.180) | (0.183) | (0.177) | (0.183) |  |
| lnGDP | -0.080^*^ | -0.072 | -0.063 | -0.068 |  | 0.054 | 0.065 | 0.038 | 0.069 |  |
|  | (0.043) | (0.047) | (0.042) | (0.047) |  | (0.062) | (0.068) | (0.061) | (0.068) |  |
| PropofIndustry | 0.135 | 0.147 | 0.141 | 0.247 |  | -0.182 | -0.238 | 0.039 | -0.142 |  |
|  | (0.212) | (0.221) | (0.197) | (0.217) |  | (0.315) | (0.329) | (0.295) | (0.320) |  |
| lnDisposableIncome | 0.124 | 0.139 | 0.050 | 0.132 |  | 0.866^***^ | 0.847^***^ | 1.007^***^ | 0.842^***^ |  |
|  | (0.166) | (0.170) | (0.161) | (0.170) |  | (0.250) | (0.257) | (0.245) | (0.257) |  |
| lnPopDens | -0.041 | -0.042 | -0.048 | -0.034 |  | -0.034 | -0.034 | -0.032 | -0.029 |  |
|  | (0.032) | (0.033) | (0.032) | (0.032) |  | (0.047) | (0.048) | (0.046) | (0.047) |  |
| DEM | 0.000 | 0.000 | 0.000 | 0.000 |  | 0.000 | 0.000 | 0.000 | 0.000 |  |
|  | (0.000) | (0.000) | (0.000) | (0.000) |  | (0.000) | (0.000) | (0.000) | (0.000) |  |
| RH | 1.383 | 1.570 | 1.597 | 1.970^*^ |  | 5.424^***^ | 5.278^***^ | 5.985^***^ | 5.719^***^ |  |
|  | (0.983) | (1.039) | (0.981) | (1.047) |  | (1.437) | (1.552) | (1.440) | (1.555) |  |
| TempAve | -0.008 | -0.009 | -0.013 | -0.010 |  | -0.019 | -0.019 | -0.030^*^ | -0.022 |  |
|  | (0.010) | (0.011) | (0.010) | (0.010) |  | (0.015) | (0.016) | (0.015) | (0.016) |  |
| PM_2.5_ | 0.012^***^ | 0.013^***^ | 0.016^***^ | 0.016^***^ |  | 0.024^***^ | 0.024^***^ | 0.026^***^ | 0.029^***^ |  |
|  | (0.005) | (0.005) | (0.005) | (0.005) |  | (0.007) | (0.007) | (0.007) | (0.007) |  |
| NDVI | -1.596^***^ | -1.633^***^ | -1.513^***^ | -1.475^***^ |  | -0.850 | -0.796 | -0.970 | -0.612 |  |
|  | (0.460) | (0.461) | (0.454) | (0.462) |  | (0.689) | (0.689) | (0.675) | (0.682) |  |
| SunshineHours | 0.000 | 0.000 | 0.000 | 0.000 |  | 0.000^***^ | 0.000^***^ | 0.000^***^ | 0.001^***^ |  |
|  | (0.000) | (0.000) | (0.000) | (0.000) |  | (0.000) | (0.000) | (0.000) | (0.000) |  |
| _cons | 0.955 | 0.471 | 1.044 | -0.221 |  | 7.816^***^ | 8.192^***^ | 6.206^**^ | 7.244^**^ |  |
|  | (1.920) | (2.052) | (1.876) | (2.046) |  | (2.784) | (3.036) | (2.762) | (3.046) |  |
| *N* | 8538 | 8272 | 8897 | 8272 |  | 8503 | 8238 | 8860 | 8238 |  |
| Pseudo/adj. *R*^2^ | 0.132 | 0.131 | 0.133 | 0.132 |  | 0.069 | 0.065 | 0.072 | 0.065 |  |

Note: Standard errors clustered at the county/district level are in parentheses. *** p < 0.01, ** p < 0.05, * p < 0.1.

**Table S8** Regression analysis of the relationship between urban greening and physical and mental health (Robustness test 3)

| Model | Health_physical | | | |  | Health_mental | | | |
| --- | --- | --- | --- | --- | --- | --- | --- | --- | --- |
|  | (a_R3) | (b_R3) | (c_R3) | (d_R3) |  | (e_R3) | (f_R3) | (g_R3) | (h_R3) |
| **GC** | **0.004^*^** |  |  |  |  | **0.004** |  |  |  |
|  | (0.002) |  |  |  |  | (0.007) |  |  |  |
| **GS** |  | **0.004^**^** |  |  |  |  | **0.002** |  |  |
|  |  | (0.002) |  |  |  |  | (0.007) |  |  |
| **PGS** |  |  | **0.012^***^** |  |  |  |  | **0.014** |  |
|  |  |  | (0.003) |  |  |  |  | (0.010) |  |
| **PGSRatio** |  |  |  | **0.002^**^** |  |  |  |  | **0.006^*^** |
|  |  |  |  | (0.001) |  |  |  |  | (0.003) |
| Age | -0.022^***^ | -0.022^***^ | -0.021^***^ | -0.022^***^ |  | -0.005 | -0.006^*^ | -0.005 | -0.006 |
|  | (0.001) | (0.001) | (0.001) | (0.001) |  | (0.003) | (0.004) | (0.003) | (0.004) |
| 0.Gender | 0.000 | 0.000 | 0.000 | 0.000 |  | 0.000 | 0.000 | 0.000 | 0.000 |
|  | (.) | (.) | (.) | (.) |  | (.) | (.) | (.) | (.) |
| 1.Gender | 0.151^***^ | 0.155^***^ | 0.150^***^ | 0.155^***^ |  | 0.278^**^ | 0.294^**^ | 0.295^**^ | 0.296^**^ |
|  | (0.029) | (0.030) | (0.029) | (0.030) |  | (0.118) | (0.120) | (0.118) | (0.120) |
| Education | 0.004 | 0.003 | 0.003 | 0.003 |  | 0.079^***^ | 0.077^***^ | 0.075^***^ | 0.077^***^ |
|  | (0.003) | (0.003) | (0.003) | (0.003) |  | (0.012) | (0.012) | (0.012) | (0.012) |
| 0.Marriage | 0.000 | 0.000 | 0.000 | 0.000 |  | 0.000 | 0.000 | 0.000 | 0.000 |
|  | (.) | (.) | (.) | (.) |  | (.) | (.) | (.) | (.) |
| 1.Marriage | -0.009 | -0.011 | -0.011 | -0.012 |  | 0.521^***^ | 0.518^***^ | 0.541^***^ | 0.516^***^ |
|  | (0.031) | (0.032) | (0.031) | (0.032) |  | (0.106) | (0.107) | (0.105) | (0.108) |
| Income | 0.211^***^ | 0.212^***^ | 0.209^***^ | 0.212^***^ |  | 0.417^***^ | 0.412^***^ | 0.415^***^ | 0.410^***^ |
|  | (0.013) | (0.013) | (0.012) | (0.013) |  | (0.048) | (0.048) | (0.047) | (0.048) |
| 0.Smoke | 0.000 | 0.000 | 0.000 | 0.000 |  | 0.000 | 0.000 | 0.000 | 0.000 |
|  | (.) | (.) | (.) | (.) |  | (.) | (.) | (.) | (.) |
| 1.Smoke | 0.051 | 0.042 | 0.047 | 0.040 |  | -0.194 | -0.199 | -0.209 | -0.208 |
|  | (0.032) | (0.033) | (0.032) | (0.033) |  | (0.130) | (0.132) | (0.130) | (0.132) |
| 0.Drink | 0.000 | 0.000 | 0.000 | 0.000 |  | 0.000 | 0.000 | 0.000 | 0.000 |
|  | (.) | (.) | (.) | (.) |  | (.) | (.) | (.) | (.) |
| 1.Drink | 0.173^***^ | 0.179^***^ | 0.191^***^ | 0.183^***^ |  | 0.156 | 0.111 | 0.135 | 0.119 |
|  | (0.037) | (0.038) | (0.036) | (0.038) |  | (0.155) | (0.156) | (0.153) | (0.156) |
| Exercise | 0.013^***^ | 0.012^**^ | 0.013^***^ | 0.013^**^ |  | 0.047^**^ | 0.042^**^ | 0.047^**^ | 0.043^**^ |
|  | (0.005) | (0.005) | (0.005) | (0.005) |  | (0.020) | (0.020) | (0.020) | (0.020) |
| 0.Overweight | 0.000 | 0.000 | 0.000 | 0.000 |  | 0.000 | 0.000 | 0.000 | 0.000 |
|  | (.) | (.) | (.) | (.) |  | (.) | (.) | (.) | (.) |
| 1.Overweight | -0.329^***^ | -0.331^***^ | -0.330^***^ | -0.330^***^ |  | -0.074 | -0.078 | -0.065 | -0.075 |
|  | (0.042) | (0.043) | (0.041) | (0.043) |  | (0.153) | (0.155) | (0.151) | (0.155) |
| 0.Underweight | 0.000 | 0.000 | 0.000 | 0.000 |  | 0.000 | 0.000 | 0.000 | 0.000 |
|  | (.) | (.) | (.) | (.) |  | (.) | (.) | (.) | (.) |
| 1.Underweight | -0.139^***^ | -0.139^***^ | -0.139^***^ | -0.143^***^ |  | 0.103 | 0.077 | 0.114 | 0.066 |
|  | (0.052) | (0.052) | (0.051) | (0.052) |  | (0.182) | (0.182) | (0.181) | (0.182) |
| lnGDP | -0.023 | -0.013 | -0.015 | -0.008 |  | 0.031 | 0.067 | 0.033 | 0.072 |
|  | (0.017) | (0.019) | (0.017) | (0.019) |  | (0.071) | (0.074) | (0.072) | (0.074) |
| PropofIndustry | 0.010 | -0.002 | 0.031 | 0.054 |  | 0.112 | 0.089 | 0.215 | 0.138 |
|  | (0.085) | (0.089) | (0.080) | (0.087) |  | (0.335) | (0.338) | (0.319) | (0.328) |
| lnDisposableIncome | 0.014 | 0.010 | -0.017 | -0.003 |  | 0.481^*^ | 0.460^*^ | 0.547^**^ | 0.430^*^ |
|  | (0.068) | (0.068) | (0.066) | (0.068) |  | (0.257) | (0.261) | (0.252) | (0.260) |
| lnPopDens | -0.024^*^ | -0.026^*^ | -0.026^**^ | -0.021 |  | -0.030 | -0.034 | -0.026 | -0.032 |
|  | (0.013) | (0.013) | (0.013) | (0.013) |  | (0.048) | (0.048) | (0.048) | (0.048) |
| DEM | -0.000 | 0.000 | -0.000 | -0.000 |  | -0.000 | -0.000 | -0.000 | -0.000 |
|  | (0.000) | (0.000) | (0.000) | (0.000) |  | (0.000) | (0.000) | (0.000) | (0.000) |
| RH | 0.294 | 0.318 | 0.293 | 0.248 |  | 0.827 | 0.786 | 1.153 | 0.732 |
|  | (0.321) | (0.324) | (0.319) | (0.323) |  | (1.288) | (1.298) | (1.274) | (1.291) |
| TempAve | -0.007^*^ | -0.008^**^ | -0.009^**^ | -0.009^**^ |  | -0.010 | -0.012 | -0.015 | -0.015 |
|  | (0.004) | (0.004) | (0.004) | (0.004) |  | (0.013) | (0.013) | (0.013) | (0.013) |
| PM_2.5_ | 0.004^**^ | 0.004^***^ | 0.006^***^ | 0.005^***^ |  | 0.007 | 0.007 | 0.009 | 0.009 |
|  | (0.002) | (0.002) | (0.002) | (0.002) |  | (0.006) | (0.006) | (0.006) | (0.006) |
| NDVI | -0.480^***^ | -0.474^**^ | -0.414^**^ | -0.374^**^ |  | 0.333 | 0.414 | 0.319 | 0.527 |
|  | (0.185) | (0.186) | (0.182) | (0.184) |  | (0.719) | (0.721) | (0.710) | (0.718) |
| _cons | 3.542^***^ | 3.434^***^ | 3.712^***^ | 3.565^***^ |  | -5.919^**^ | -5.999^**^ | -6.841^***^ | -5.919^**^ |
|  | (0.620) | (0.631) | (0.604) | (0.627) |  | (2.300) | (2.374) | (2.226) | (2.333) |
| *N* | 8538 | 8272 | 8897 | 8272 |  | 8503 | 8238 | 8860 | 8238 |
| adj./Pseudo *R*^2^ | 0.129 | 0.128 | 0.131 | 0.128 |  | 0.063 | 0.062 | 0.063 | 0.062 |

Note: Standard errors clustered at the county/district level are in parentheses. *** p < 0.01, ** p < 0.05, * p < 0.1.

**Table S9** Regression analysis of the relationship between urban greening and physical health (Robustness test 4)

|  | Health_hospitalized | | | |
| --- | --- | --- | --- | --- |
|  | (a_R4) | (b_R4) | (c_R4) | (d_R4) |
| **GC** | **0.006** |  |  |  |
|  | (0.007) |  |  |  |
| **GS** |  | **-0.001** |  |  |
|  |  | (0.006) |  |  |
| **PGS** |  |  | **-0.023^**^** |  |
|  |  |  | (0.009) |  |
| **PGSRatio** |  |  |  | **-0.009^***^** |
|  |  |  |  | (0.003) |
| Age | 0.036^***^ | 0.037^***^ | 0.038^***^ | 0.037^***^ |
|  | (0.003) | (0.003) | (0.003) | (0.003) |
| 0.Gender | 0.000 | 0.000 | 0.000 | 0.000 |
|  | (.) | (.) | (.) | (.) |
| 1.Gender | -0.073 | -0.087 | -0.086 | -0.089 |
|  | (0.098) | (0.099) | (0.096) | (0.099) |
| Education | -0.021^**^ | -0.021^**^ | -0.017^*^ | -0.020^**^ |
|  | (0.010) | (0.010) | (0.009) | (0.010) |
| 0.Marriage | 0.000 | 0.000 | 0.000 | 0.000 |
|  | (.) | (.) | (.) | (.) |
| 1.Marriage | 0.046 | 0.045 | 0.048 | 0.049 |
|  | (0.105) | (0.106) | (0.104) | (0.106) |
| Income | -0.102^***^ | -0.091^**^ | -0.080^**^ | -0.090^**^ |
|  | (0.037) | (0.037) | (0.036) | (0.037) |
| 0.Smoke | 0.000 | 0.000 | 0.000 | 0.000 |
|  | (.) | (.) | (.) | (.) |
| 1.Smoke | -0.005 | 0.007 | -0.030 | 0.016 |
|  | (0.110) | (0.111) | (0.109) | (0.112) |
| 0.Drink | 0.000 | 0.000 | 0.000 | 0.000 |
|  | (.) | (.) | (.) | (.) |
| 1.Drink | -0.646^***^ | -0.641^***^ | -0.621^***^ | -0.656^***^ |
|  | (0.140) | (0.142) | (0.136) | (0.142) |
| Exercise | 0.025^*^ | 0.025^*^ | 0.024 | 0.025 |
|  | (0.015) | (0.015) | (0.015) | (0.015) |
| 0.Overweight | 0.000 | 0.000 | 0.000 | 0.000 |
|  | (.) | (.) | (.) | (.) |
| 1.Overweight | 0.112 | 0.105 | 0.089 | 0.098 |
|  | (0.130) | (0.132) | (0.127) | (0.133) |
| 0.Underweight | 0.000 | 0.000 | 0.000 | 0.000 |
|  | (.) | (.) | (.) | (.) |
| 1.Underweight | 0.512^***^ | 0.515^***^ | 0.542^***^ | 0.533^***^ |
|  | (0.146) | (0.146) | (0.144) | (0.147) |
| lnGDP | -0.004 | 0.026 | -0.002 | 0.019 |
|  | (0.058) | (0.062) | (0.057) | (0.062) |
| PropofIndustry | -0.036 | -0.034 | 0.010 | -0.105 |
|  | (0.283) | (0.295) | (0.264) | (0.289) |
| lnDisposableIncome | -0.130 | -0.098 | -0.136 | -0.069 |
|  | (0.237) | (0.233) | (0.226) | (0.231) |
| lnPopDens | 0.055 | 0.058 | 0.072 | 0.056 |
|  | (0.045) | (0.045) | (0.044) | (0.044) |
| DEM | 0.001^***^ | 0.001^***^ | 0.001^***^ | 0.001^***^ |
|  | (0.000) | (0.000) | (0.000) | (0.000) |
| RH | 2.116^*^ | 2.085^*^ | 1.610 | 2.069^*^ |
|  | (1.134) | (1.117) | (1.111) | (1.115) |
| TempAve | 0.006 | 0.004 | 0.012 | 0.009 |
|  | (0.013) | (0.013) | (0.013) | (0.013) |
| PM_2.5_ | 0.017^***^ | 0.017^***^ | 0.014^***^ | 0.014^***^ |
|  | (0.005) | (0.005) | (0.005) | (0.005) |
| NDVI | 0.188 | 0.294 | 0.456 | 0.136 |
|  | (0.643) | (0.630) | (0.630) | (0.631) |
| _cons | -5.336^**^ | -5.873^***^ | -4.831^**^ | -5.713^***^ |
|  | (2.139) | (2.135) | (2.030) | (2.082) |
| *N* | 8538 | 8272 | 8897 | 8272 |
| Pseudo *R*^2^ | 0.065 | 0.066 | 0.067 | 0.068 |

Note: Standard errors clustered at the county/district level are in parentheses. *** p < 0.01, ** p < 0.05, * p < 0.1.

**Table S10** Regression analysis of the relationship between urban greening and physical and mental health (Robustness test 5)

| Model | Health_physical | | | |  | Health_mental | | | |  |
| --- | --- | --- | --- | --- | --- | --- | --- | --- | --- | --- |
|  | (a_R5) | (b_R5) | (c_R5) | (d_R5) |  | (e_R5) | (f_R5) | (g_R5) | (h_R5) |  |
| **GC** | **0.003** |  |  |  |  | **0.014^***^** |  |  |  |  |
|  | (0.004) |  |  |  |  | (0.005) |  |  |  |  |
| **GS** |  | **0.005** |  |  |  |  | **0.020^***^** |  |  |  |
|  |  | (0.004) |  |  |  |  | (0.005) |  |  |  |
| **PGS** |  |  | **0.010^*^** |  |  |  |  | **0.028^***^** |  |  |
|  |  |  | (0.006) |  |  |  |  | (0.009) |  |  |
| **PGSRatio** |  |  |  | **0.004^***^** |  |  |  |  | **0.004^**^** |  |
|  |  |  |  | (0.002) |  |  |  |  | (0.002) |  |
| Age | -0.036^***^ | -0.035^***^ | -0.036^***^ | -0.035^***^ |  | 0.005^*^ | 0.005^*^ | 0.004 | 0.005^*^ |  |
|  | (0.002) | (0.002) | (0.002) | (0.002) |  | (0.003) | (0.003) | (0.003) | (0.003) |  |
| 0.Gender | 0.000 | 0.000 | 0.000 | 0.000 |  | 0.000 | 0.000 | 0.000 | 0.000 |  |
|  | (.) | (.) | (.) | (.) |  | (.) | (.) | (.) | (.) |  |
| 1.Gender | 0.158^***^ | 0.162^***^ | 0.154^***^ | 0.161^***^ |  | 0.616^***^ | 0.607^***^ | 0.636^***^ | 0.646^***^ |  |
|  | (0.061) | (0.062) | (0.059) | (0.062) |  | (0.088) | (0.090) | (0.086) | (0.086) |  |
| Education | 0.045^***^ | 0.045^***^ | 0.045^***^ | 0.046^***^ |  | 0.034^***^ | 0.038^***^ | 0.034^***^ | 0.038^***^ |  |
|  | (0.006) | (0.006) | (0.006) | (0.006) |  | (0.009) | (0.010) | (0.009) | (0.009) |  |
| 0.Marriage | 0.000 | 0.000 | 0.000 | 0.000 |  | 0.000 | 0.000 | 0.000 | 0.000 |  |
|  | (.) | (.) | (.) | (.) |  | (.) | (.) | (.) | (.) |  |
| 1.Marriage | -0.194^***^ | -0.174^**^ | -0.178^***^ | -0.167^**^ |  | 0.465^***^ | 0.437^***^ | 0.483^***^ | 0.398^***^ |  |
|  | (0.070) | (0.072) | (0.069) | (0.072) |  | (0.102) | (0.106) | (0.100) | (0.101) |  |
| Income | 0.276^***^ | 0.273^***^ | 0.271^***^ | 0.272^***^ |  | 0.421^***^ | 0.425^***^ | 0.415^***^ | 0.435^***^ |  |
|  | (0.023) | (0.024) | (0.023) | (0.024) |  | (0.035) | (0.036) | (0.035) | (0.035) |  |
| 0.Smoke | 0.000 | 0.000 | 0.000 | 0.000 |  | 0.000 | 0.000 | 0.000 | 0.000 |  |
|  | (.) | (.) | (.) | (.) |  | (.) | (.) | (.) | (.) |  |
| 1.Smoke | 0.118^*^ | 0.107 | 0.121^*^ | 0.107 |  | -0.047 | -0.069 | -0.074 | -0.109 |  |
|  | (0.066) | (0.067) | (0.065) | (0.067) |  | (0.092) | (0.095) | (0.090) | (0.091) |  |
| 0.Drink | 0.000 | 0.000 | 0.000 | 0.000 |  | 0.000 | 0.000 | 0.000 | 0.000 |  |
|  | (.) | (.) | (.) | (.) |  | (.) | (.) | (.) | (.) |  |
| 1.Drink | 0.223^***^ | 0.190^***^ | 0.239^***^ | 0.194^***^ |  | 0.236^**^ | 0.231^**^ | 0.247^***^ | 0.194^**^ |  |
|  | (0.071) | (0.072) | (0.070) | (0.072) |  | (0.096) | (0.099) | (0.094) | (0.096) |  |
| Exercise | 0.016^**^ | 0.019^**^ | 0.017^**^ | 0.019^**^ |  | 0.086^***^ | 0.083^***^ | 0.086^***^ | 0.079^***^ |  |
|  | (0.007) | (0.008) | (0.007) | (0.008) |  | (0.011) | (0.011) | (0.011) | (0.011) |  |
| 0.Overweight | 0.000 | 0.000 | 0.000 | 0.000 |  | 0.000 | 0.000 | 0.000 | 0.000 |  |
|  | (.) | (.) | (.) | (.) |  | (.) | (.) | (.) | (.) |  |
| 1.Overweight | -0.229^***^ | -0.229^***^ | -0.245^***^ | -0.231^***^ |  | 0.223^*^ | 0.210^*^ | 0.221^*^ | 0.214^*^ |  |
|  | (0.081) | (0.083) | (0.079) | (0.083) |  | (0.118) | (0.121) | (0.115) | (0.115) |  |
| 0.Underweight | 0.000 | 0.000 | 0.000 | 0.000 |  | 0.000 | 0.000 | 0.000 | 0.000 |  |
|  | (.) | (.) | (.) | (.) |  | (.) | (.) | (.) | (.) |  |
| 1.Underweight | -0.286^***^ | -0.286^***^ | -0.281^***^ | -0.289^***^ |  | -0.179 | -0.256 | -0.181 | -0.245 |  |
|  | (0.098) | (0.101) | (0.097) | (0.101) |  | (0.157) | (0.162) | (0.155) | (0.152) |  |
| lnGDP | 0.046 | 0.010 | 0.042 | 0.035 |  | -0.080 | -0.158^***^ | -0.072 | -0.096^*^ |  |
|  | (0.036) | (0.040) | (0.035) | (0.039) |  | (0.054) | (0.060) | (0.052) | (0.053) |  |
| PropofIndustry | 0.021 | 0.086 | 0.008 | 0.177 |  | 0.164 | 0.348 | 0.370 | 0.410 |  |
|  | (0.187) | (0.198) | (0.179) | (0.196) |  | (0.273) | (0.290) | (0.263) | (0.277) |  |
| lnDisposableIncome | -0.192 | -0.166 | -0.156 | -0.233 |  | 0.578^***^ | 0.574^***^ | 0.643^***^ | 0.542^***^ |  |
|  | (0.142) | (0.143) | (0.138) | (0.143) |  | (0.201) | (0.203) | (0.195) | (0.195) |  |
| lnPopDens | -0.046 | -0.042 | -0.036 | -0.025 |  | 0.046 | 0.062 | 0.076^*^ | 0.132^***^ |  |
|  | (0.029) | (0.029) | (0.029) | (0.030) |  | (0.046) | (0.046) | (0.044) | (0.041) |  |
| DEM | 0.000 | 0.000 | 0.000 | 0.000 |  | -0.000^***^ | -0.000^***^ | -0.000^***^ | -0.000^***^ |  |
|  | (0.000) | (0.000) | (0.000) | (0.000) |  | (0.000) | (0.000) | (0.000) | (0.000) |  |
| RH | 0.510 | 0.525 | 0.530 | 0.378 |  | 2.661^***^ | 2.439^**^ | 2.637^***^ | 0.718 |  |
|  | (0.647) | (0.653) | (0.639) | (0.644) |  | (0.983) | (1.015) | (0.978) | (0.913) |  |
| TempAve | -0.002 | -0.001 | -0.003 | -0.001 |  | -0.019 | -0.015 | -0.019 | -0.019^*^ |  |
|  | (0.008) | (0.008) | (0.008) | (0.008) |  | (0.012) | (0.012) | (0.012) | (0.011) |  |
| PM_2.5_ | 0.010^***^ | 0.010^***^ | 0.011^***^ | 0.012^***^ |  | 0.009^**^ | 0.008^**^ | 0.011^***^ | 0.008^**^ |  |
|  | (0.003) | (0.003) | (0.003) | (0.003) |  | (0.004) | (0.004) | (0.004) | (0.004) |  |
| NDVI | -0.862^**^ | -0.931^**^ | -0.844^**^ | -0.704^*^ |  | -1.612^**^ | -1.566^**^ | -1.553^**^ | 0.067 |  |
|  | (0.416) | (0.416) | (0.405) | (0.407) |  | (0.643) | (0.657) | (0.630) | (0.569) |  |
| _cons | 2.668^**^ | 2.770^**^ | 2.259^*^ | 2.931^**^ |  | 17.626^***^ | 18.585^***^ | 16.687^***^ | 18.539^***^ |  |
|  | (1.227) | (1.258) | (1.195) | (1.254) |  | (1.700) | (1.757) | (1.666) | (1.711) |  |
| *N* | 9834 | 9282 | 10145 | 9282 |  | 8648 | 8118 | 8950 | 8919 |  |
| Pseudo/adj. *R*^2^ | 0.088 | 0.086 | 0.089 | 0.086 |  | 0.063 | 0.060 | 0.065 | 0.056 |  |

Note: Standard errors clustered at the county/district level are in parentheses. *** p < 0.01, ** p < 0.05, * p < 0.1.
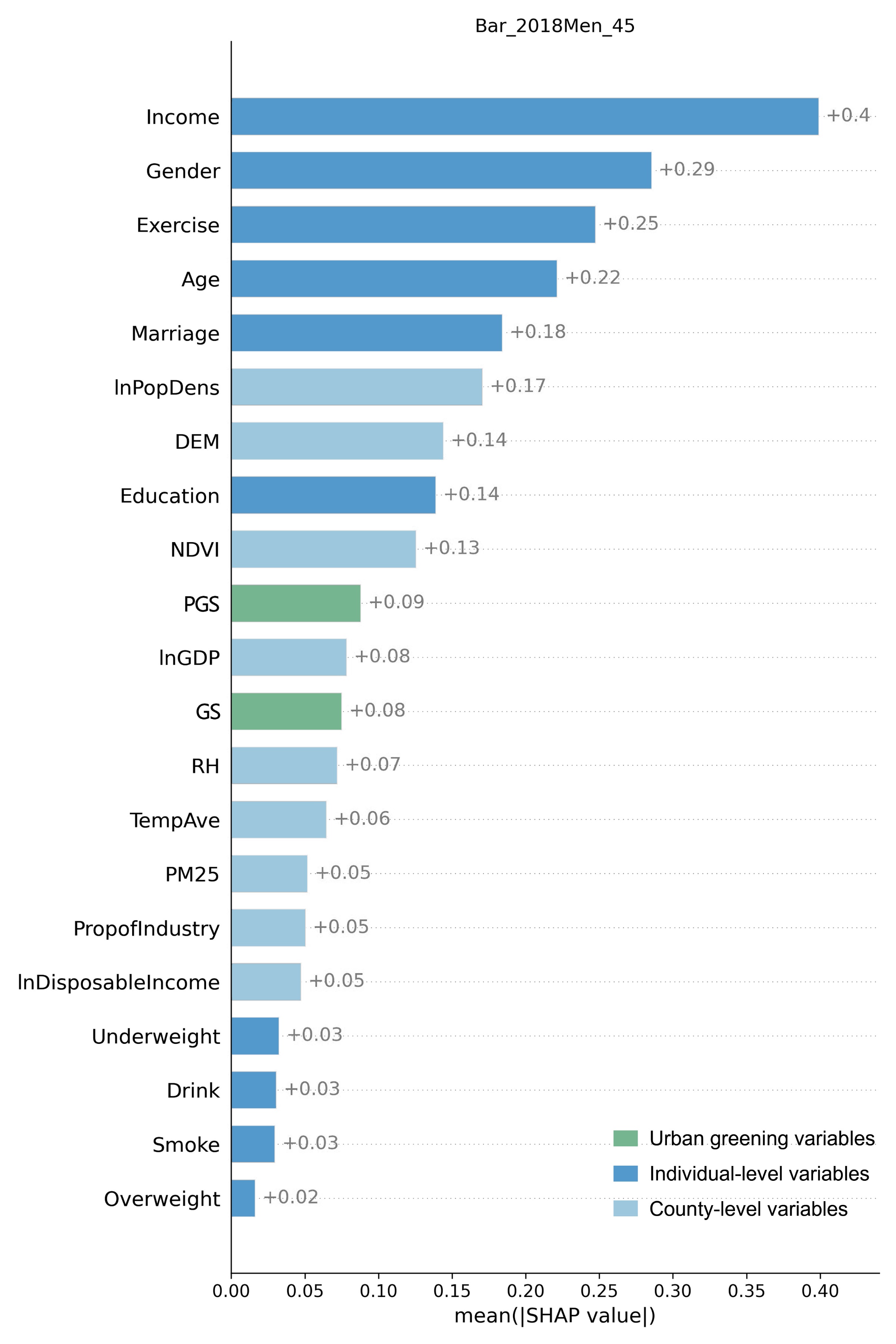

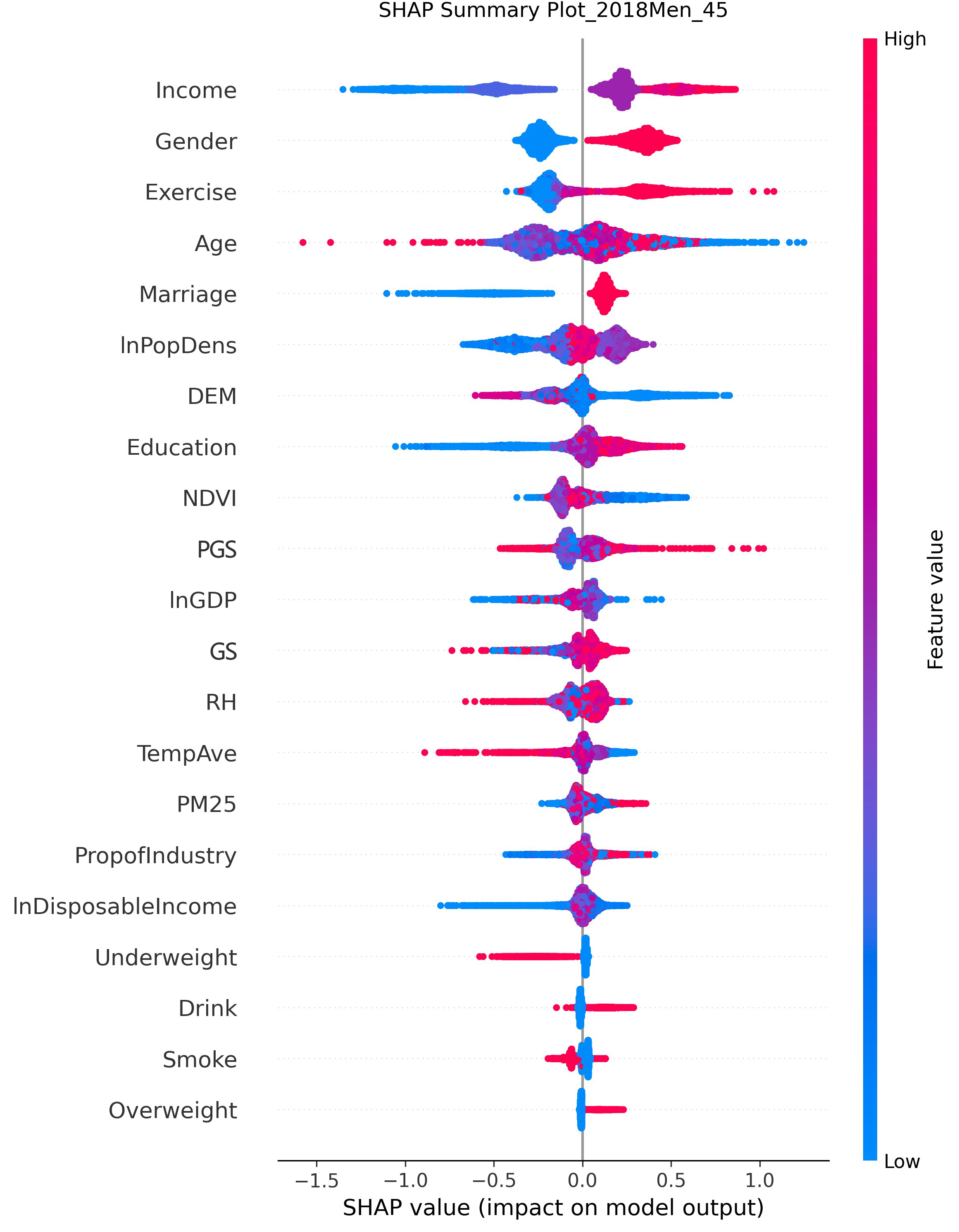


**Fig. S1.** Relative importance of GS and PGS on mental health using data in 2018 (Robustness test 5).

**Appendix II**

Following are the reasons why we consider the item “How would you rate your health status?” to be a physical health indicator instead of a general health indicator.

1. Sequence of Question Items and Module Setting

This item is the fifth item in **Part P. Health**. The first four items ask about the respondent's height, weight, weight at birth, and usual use of hands. The items following this item ask, in order, about changes in respondents’ health status, whether they felt any physical discomfort and what kind, the severity of the symptoms, visits to the doctor, whether they have any doctor-diagnosed chronic disease and what kind, the most serious disease they’ve ever had. As can be seen, the items before and after this item are essentially questions about physical health. Due to cognitive inertia, respondents tend to respond to this item based on their physical health. Items related to *Medical Expenditure* and *Medical Insurance* are also included in this part. In addition, the CFPS questionnaire has specific items (the CES-D scale) on mental health conditions, which are included in **Part Q. Behavior and Mental Status**.

1. Influence of cultural and social background

The term ‘health’ in the Chinese context is often directly associated with physiological conditions such as illness and physical strength in daily communication. Public perceptions of health tend to be more biased towards physiological dimensions, such as being free of disease and having normal bodily functions. Mental health is more often categorized as a separate concept, such as anxiety, mood, and stress. Compared to Western countries, the concept of mental health has been popularized relatively late in China, and although awareness has increased in recent years, public awareness of mental health issues remains low (State Council of the People’s Republic of China, 2019). If ‘mental’ is not explicitly mentioned in the question, most people may not take it into account.

State Council of the People’s Republic of China. (2019). Healthy China Initiative 2019-2030. Retrieved from <https://www.gov.cn/xinwen/2019-07/15/content_5409694.htm>.

1. Cross-validation

We used regression and correlation analyses to quantify the contribution of physical and mental health condition-related items to the target item. The R^2^ values and correlation coefficients for the items “During the past two weeks, have you felt any physical discomfort?”, “How serious do you think the symptom is?”, “During the past six months, have you had any doctor-diagnosed chronic disease?”, etc. were between 0.12-0.15 and 0.35-0.40, respectively. The R^2^ values and correlation coefficients for the 8 items of the CES-D scale (mental condition scale, see Appendix I) were only around 0.02-0.03 and 0.10-0.20, respectively. The items related to physical health conditions have more explanatory power than the CES-D scale, which supports our use of this item as a physical health indicator.

1. Empirical study

An empirical study on the elders in Shanghai found that the contribution of mental health to self-rated health was 8.5%, while the contribution of physical conditions and chronic diseases were 33%. This suggests that SRH reflects physical health conditions much more than mental health conditions. However, this gap may be narrowing among younger residents.

Dong, W., Wan, J., Xu, Y. *et al.*  (2017). Determinants of self-rated health among Shanghai elders: a cross-sectional study. *BMC Public Health* 17, 807.

Based on the above considerations, we consider that this item is biased towards reflecting respondents’ self-rated physical health. A small number of respondents may consider mental health in combination. However, physical health and mental health are two closely related dimensions of health, and respondents may also be influenced by their physical health when answering the mental health questions. As physical health is difficult to quantify and can be influenced by numerous factors, we used this item as a general assessment of respondents' physical health conditions.
